# Proteomic Detection of Non-Annotated Protein-Coding Genes in *Pseudomonas fluorescens* Pf0-1

**DOI:** 10.1371/journal.pone.0008455

**Published:** 2009-12-24

**Authors:** Wook Kim, Mark W. Silby, Sam O. Purvine, Julie S. Nicoll, Kim K. Hixson, Matt Monroe, Carrie D. Nicora, Mary S. Lipton, Stuart B. Levy

**Affiliations:** 1 Center for Adaptation Genetics and Drug Resistance and Department of Molecular Biology and Microbiology, Tufts University School of Medicine, Boston, Massachusetts, United States of America; 2 Pacific Northwest National Laboratory, Richland, Washington, United States of America; Max Planck Institute for Evolutionary Anthropology, Germany

## Abstract

Genome sequences are annotated by computational prediction of coding sequences, followed by similarity searches such as BLAST, which provide a layer of possible functional information. While the existence of processes such as alternative splicing complicates matters for eukaryote genomes, the view of bacterial genomes as a linear series of closely spaced genes leads to the assumption that computational annotations that predict such arrangements completely describe the coding capacity of bacterial genomes. We undertook a proteomic study to identify proteins expressed by *Pseudomonas fluorescens* Pf0-1 from genes that were not predicted during the genome annotation. Mapping peptides to the Pf0-1 genome sequence identified sixteen non-annotated protein-coding regions, of which nine were antisense to predicted genes, six were intergenic, and one read in the same direction as an annotated gene but in a different frame. The expression of all but one of the newly discovered genes was verified by RT-PCR. Few clues as to the function of the new genes were gleaned from informatic analyses, but potential orthologs in other *Pseudomonas* genomes were identified for eight of the new genes. The 16 newly identified genes improve the quality of the Pf0-1 genome annotation, and the detection of antisense protein-coding genes indicates the under-appreciated complexity of bacterial genome organization.

## Introduction

Across all microbial genomes, organization of genes in a genome is less modular than the typical portrayal of a linear series of discrete regulatory and coding regions; the density of encoded information is amplified as neighboring genes can share common nucleotides, arrangements that may have been selected because of the benefits of compressing genetic information, or because of a regulatory relationship between the overlapping sequences [1]-[3]. While major advances have been made to ensure the quality of the genome sequencing process and the resulting products, there is room for improvement of the current annotation process. A combination of gene modeling programs has been utilized for annotation, such as Generation, Glimmer, Critica, and more recently, Prodigal (http://genome.ornl.gov/microbial/notes.html), often augmented by manual annotation. The final product is bound to contain mis-annotated genes since it is dependent on the individual parameters and settings of the chosen models, and published literature used by human annotators. In general, only short overlaps between the ends of coding sequences are considered during both computational and manual annotation efforts. While the algorithms of the gene models continue to be developed, their intrinsic dependence on history pushes the bias towards previous knowledge, potentially propagating errors and omissions in subsequent annotations. For example, genome sequence annotations seldom include overlapping genes where one member of the pair is fully embedded within the coding sequence of the other. Continued development and application of post-annotation quality control programs like MisPred [4] should help to reduce the frequency of errors and their subsequent transmission, as will experimental verification of novel gene arrangements [e.g. 5].

To fully appreciate the functional genome of a given organism and to improve gene annotation, it is necessary to go beyond the predictive step into experimentation. For a model organism like *Escherichia coli* K-12, a tremendous amount of experimental data have accumulated over time, providing a layer of experimentally verified functional data in the genome annotation [6]. However, for less widely studied organisms, experimental data are usually absent from genome annotations. In these cases, high-throughput expression technologies can provide verification that annotated genes are expressed, thus improving the accuracy and reliability of the annotation. Largely because of the continued technical advances in the field of mass spectrometry, direct determination of proteins produced by an organism is becoming increasingly feasible, providing an opportunity to not only confirm the existence of genes predicted in genome annotations, but also to identify previously non-predicted proteins. A recent analysis of the *Arabidopsis* proteome identified greater than 18,000 peptides that did not correspond to the current annotation, leading to the refinement of current gene models and the description of previously non-annotated genes [7].

We previously demonstrated the existence of a pair of antiparallel genes in *P. fluorescens* Pf0-1 that share extensive overlapping coding sequences, and both specify proteins. Only one member of the pair had been annotated in the Pf0-1 genome sequence [5]. The objective of this study was to determine whether additional non-predicted proteins are produced by Pf0-1, and to map these onto the Pf0-1 genome sequence relative to predicted genes. We present evidence for the existence of 16 non-predicted genes in Pf0-1, which can be classified into three groups relative to predicted coding sequences: intergenic, antisense, and frame shifted.

## Results and Discussion

### High Throughput Analysis of the *P. fluorescens* Pf0-1 Proteome

Our laboratory is characterizing the functional genome of *Pseudomonas fluorescens* Pf0-1 [8], with particular emphasis on the molecular mechanisms governing environmental adaptation. As part of our ongoing efforts to annotate the genome [9], we sought to generate an extensive protein expression profile by utilizing a variety of growth conditions, which would allow detection of proteins expressed under specific growth phase and nutritional conditions. In addition to the parent strain Pf0-1, the *adnA* mutant strain Pf0-2x [10] was independently grown and harvested under the same set of conditions. AdnA (FleQ) is a transcriptional regulator previously demonstrated to be required for motility, and to modulate biofilm formation and adhesion to sand particles in Pf0-1 [10]. In order to identify proteins specified by non-annotated genes, we analyzed peptide data using a stop to stop database based on the Pf0-1 genome sequence. This analysis was done rather than using the previously annotated ORFs (GenBank accession CP000094) as the search field to ensure that proteins not predicted from the current annotation would not be excluded. Greater than 106,000 peptides were identified in total, corresponding to 3769 out of 5736 annotated ORFs (∼65.7% coverage). Products encoded by the remaining ORFs may have escaped our detection because of various technical limitations associated with our experimental strategy: their low levels of expression, incompatible buffers for extractions, absence of secreted proteins, and incorrect annotations. In addition, it is likely that certain ORFs require specific growth conditions that were not replicated in our experiments, such as those that are activated only when the cells are grown in soil [11]. In comparison, a recent proteogenomic profiling of *Arabidopsis* identified c.a. 40% of the annotated genes [7].

### Non-Predicted Proteins Revealed by Proteome Analysis

The peptide set also generated hits to 239 ORFs that were not predicted to be protein coding in the current annotation (GenBank accession CP000094). Because global expression datasets are vulnerable to both false positives and negatives, we applied additional filtering rules to the 239 ORFs dataset to identify a strong subset of candidates for experimental verification: each ORF contains a major upstream start codon (ATG, GTG, and TTG); is represented by fully tryptic or partially tryptic terminal peptides carrying a minimum PeptideProphet score of 0.95; and is present in both Pf0-1 and Pf0-2x datasets. Sixteen ORFs passed the stringent criteria and were named *nov1* through *nov16* as they potentially represent novel genes not predicted in the Pf0-1 genome (Table 1).

**Table 1:** General features of *nov* genes in *P. fluorescens* Pf0-1.

*nov*	Organization^a^	Length^b^	Genome Coordinates^c^	Culture Conditions^d^	Potential SD Sequence^e^
01	overlap (opp. & par.)	195	c747558-748142	Min	ccgaacacgacgaacacgtggtgcggctgtg
02	intergenic (small par. overlap)	87	c1046719-1046979	Min/Rich-stn	acccattccgaaatgagaaaacaaaaaaatg
03	overlap (opp.)	90	c1165207-1165476	Rich-exp	acacttcataagccggcgtctcgtagggatg
04	overlap (opp.)	71	c2784682-2784894	Min/Rich-stn	taaaagcacttacaaaaggagacttcacatg
05	intergenic (par.)	98	c2950927-2951220	Min/Rich	aaattaaacatcagatgaggtgtttttcatg
06	overlap (opp. two ORFs)	178	c3059127-3059660	Min/Rich-stn	cgcgcagtacccagcggacgatgtcctggtg
07	overlap (opp.)	42	c3285610-3285735	Rich-exp	gatatcgaactgcaacagacgtccggccttg
08	overlap (opp.)	152	c3526135-3526590	Rich-exp	gcgcatcggtgtttttcaccggttcaccgtg
09	intergenic (opp./par.)	56	c3594859-3595026	Rich-stn	cacattgcgactctcaacgaggtgccttatg
10	intergenic (opp.)	62	c3774366-3774551	Min	aaagttcttcagcagacaggaggtgcctatg
11	overlap (opp. two ORFs)	155	c4134875-4135339	Min	aggacacgtaaccgcgcacggtaacgccgtg
12	intergenic (small par. overlap)	50	c4285307-4285456	Min	agttctttcaggcgggtttgatgcatgggtg
13	overlap (par.)	211	4475971-4476603	Min	acgactatgtgatcaacggcagcaagatgtg
14	overlap (opp.)	101	c4756241-4756543	Min	ccgcgacattcagtcgcagggcggttaagttg
15	overlap (opp.)	530	c4938030-4939619	Min/Rich-exp	ccgcattcgaggcgctgttggccacgtcgtg
16	intergenic (par.)	73	5264889-5265107	Min/Rich-exp	gcacttgccaagcaagaaggttttcaagatg

a opp. indicates overlap with gene coded on opposite DNA strand; par. indicates overlap with gene coded parallel, in a different frame.

b Predicted length of novel gene product (amino acids).

c In coordinates, the ‘c’ indicates the ORF is complementary to the coordinates shown. These coordinates represent the smallest potential coding sequence, defined by the first possible initiation codon upstream of the peptides.

d These are the conditions for growth of cultures from which the Nov proteins were identified. Min indicates *Pseudomonas* minimal medium; Rich indicates King's B or LB; exp indicates exponential growth phase; stn indicates stationary growth phase.

e Possible Shine-Dalgarno sequences are underlined, and in bold. Potential translation initiation codons are in bold.

The translation start codon of each *nov* ORF (*n*ORF) was arbitrarily assigned to the first potential in-frame start codon upstream from the identified peptides. Relevant attributes of the *n*ORFs are summarized in Table 1. With the exception of *nov7* and *nov13*, different variations of experimentally confirmed *E. coli* Shine-Dalgarno sequences [12], [13] were identified within the upstream region of each *n*ORF, which are consistent with expectations based on the 3′ sequence of the 16 s rRNA in Pf0-1. The *n*ORFs were dispersed widely throughout the genome, and their predicted size varied between 126 and 1590 bp; nine were smaller than 300 bp. Within the current annotation of Pf0-1 (GenBank Accession CP000094), there are more than 200 predicted coding sequences that fall below 250 bp in length, the shortest being a 75 bp ORF predicted to encode the coenzyme PQQ synthesis protein A (Pfl01_5157). Thus, it appears unlikely that the *n*ORFs escaped the initial gene hunting algorithms simply because of their relatively short lengths. We used the most recent releases of GenemarkS and Glimmer to test whether any of the *n*ORFs could be predicted by computational means. We were able to detect five of the *n*ORFs using the embedded default parameters, while the remaining 11 remained non-predicted (Table 2 and 3).

**Table 2:** Analysis of *nov* genes that overlap predicted genes in *P. fluorescens* Pf0-1.

*nov*	Predictions^a^	TBLASTN – relevant hits
01	None	*P. fluorescens* Pf-5. Identities = 80/178 (44%), Positives = 97/178 (54%), Gaps = 7/178 (3%). ORF is 301 codons long. Similar organization to *nov1*.
		Other *Pseudomonas* sp. (many interrupted by stop codons).
03	None	*P. fluorescens* SBW25. Identities = 38/88 (43%), Positives = 52/88 (59%), Gaps = 0/88 (0%). Similarity at 5′ end of 129 codon ORF antisense to PFLU_5033 and PFLU_5034.
		*P. putida* KT2440. Identities = 40/78 (51%), Positives = 51/78 (65%), Gaps = 0/78 (0%). Similarity at 5′ end of a 1371 codon ORF, antisense to PP_1037 and PP_1038
04	GenemarkS	No significant hits
06	None	No significant hits
07	None	*P. fluorescens* SBW25. Identities = 33/40 (82%), Positives = 35/40 (87%), Gaps = 0/40 (0%). Similarity at 3′ end of a 62 codon ORF.
		*P. fluorescens* Pf-5. Identities = 31/41 (75%), Positives = 34/41 (82%), Gaps = 0/41 (0%). Similarity at 3′ end of a 63 codon ORF.
		Most hits antisense to probable nucleotide sugar dehydrogenase in *Pseudomonas* sp. Same organization as *nov7*.
08	None	*P. fluorescens* SBW25. Identities = 95/152 (62%), Positives = 114/152 (75%), Gaps = 0/152 (0%). Similarity over first ¾ of a 209 codon ORF.
		Hits to ORFs opposite acetoacetyl-CoA synthase in organisms including *Pseudomonas* sp., *Rhizobium* sp., and *Brucella* sp. Some hits have stop codons.
11	None	*P. fluorescens* SBW25. Identities = 84/129 (65%), Positives = 97/129 (75%), Gaps = 0/129 (0%), over 129aa length of possible SBW25 protein.
		Hits to sequences opposite hydroxymethylglutaryl-CoA lyase in numerous organisms including *Pseudomonas* sp., *Burkholderia* sp., and *Xanthomonas* sp.
13	None	*P. fluorescens* Pf-5 and SBW25. High identity with Nov13, but lack stop codon. Result would be fusion with downstream gene in same reading frame. In each genome there is a run of Arg codons, ending with cga-cga in SBW25 and Pf-5. In Pf0-1 there is cga-tga. The tga stop codon may be from a new mutation.
		Similar *P. aeruginosa* sequences have stop codons at codon 61.
14	None	*P. fluorescens* Pf-5. Identities = 57/63 (90%), Positives = 57/63 (90%), Gaps = 0/63 (0%). Very similar sequence matches from amino acid 39 of *nov14*. Pf-5 ORF codes for 63 aa (Nov14 codes 101). Upstream two Val codons (gtt-gtc) are in a suitable location that if altered to a ‘gtg’ initiation codon could code for a nearly identical protein to Nov14.
		Numerous other *Pseudomonas* sp. show high identity. SBW25 has a stop codon, others do not.
15	None	*P. fluorescens* Pf-5. Identities = 298/491 (60%), Positives = 340/491 (69%), Gaps = 2/491 (0%). ORF is 502 codons.
		*P. fluorescens* SBW25 has a similar sequence, but it is interrupted by two stop codons. Similar sequences occur in *P. aeruginosa*. No stop codon in PA14; PAO1 interrupted by a stop codon.

a Gene prediction using GenemarkS and Glimmer.

**Table 3:** Analysis of *nov* genes located in ‘intergenic’ regions in *P. fluorescens* Pf0-1.

*nov*	Predictions^a^	TBLASTN – relevant hits	Orthologs^b^
02	GenemarkS	Best hit: *Photorhabdus asymbiotica* subsp. *asymbiotica* ATCC 43949. Identities = 50/84 (59%), Positives = 67/84 (79%), Gaps = 1/84 (1%).	colicin-pyocin immunity protein
		Also, *Photorhabdus* sp., *Yersinia* sp., and *Pseudomonas* sp.	
05	None	No significant hits	None
09	GenemarkS & Glimmer	Pf0-1 genome	None
10	GenemarkS & Glimmer	*P. fluorescens* Pf-5. Identities = 28/41 (68%), Positives = 35/41 (85%), Gaps = 0/41 (0%). Similar to first 50 codons (by clustalW2) of PFL_2158 (62 codons).	None
12	None	Pf0-1 genome	None
16	GenemarkS & Glimmer	*P. fluorescens* P5-5. Hits the predicted gene PFL_5052. Good alignment over N-terminal 45 aa, less over the remaining 28 Nov16 aa. *Pseudomonas putida* KT2440 aligns similarly. In Pf0-1, first 24aa is likely a signal sequence.	signal peptide at N-terminus
		Six hits, all in *Pseudomonas* sp. (Possibly due to putative signal sequence).	

a Gene prediction using GenemarkS and Glimmer.

b Indicates matches in GenBank for which functional predictions have been made.

Although it has been argued that most hypothetical ORFs that are predicted to encode short-length proteins may in fact be mis-annotated random stretches of DNA [14], there is increasing evidence that many do indeed express proteins [15]. Some small proteins have been shown to play important physiological roles, such as sporulation in *Bacillus subtilis* [16] and chromosome segregation in *Saccharomyces cerevisiae* [17]. In addition, our laboratory identified a 41 amino acid protein (Blr) which confers intrinsic resistance to a broad spectrum of β-lactam antibiotics upon *Escherichia coli* [18]. The *blr* gene had escaped annotation, and was located between two annotated genes [18].

### Novel Overlapping Genes in Pf0-1

**Figure 1:**
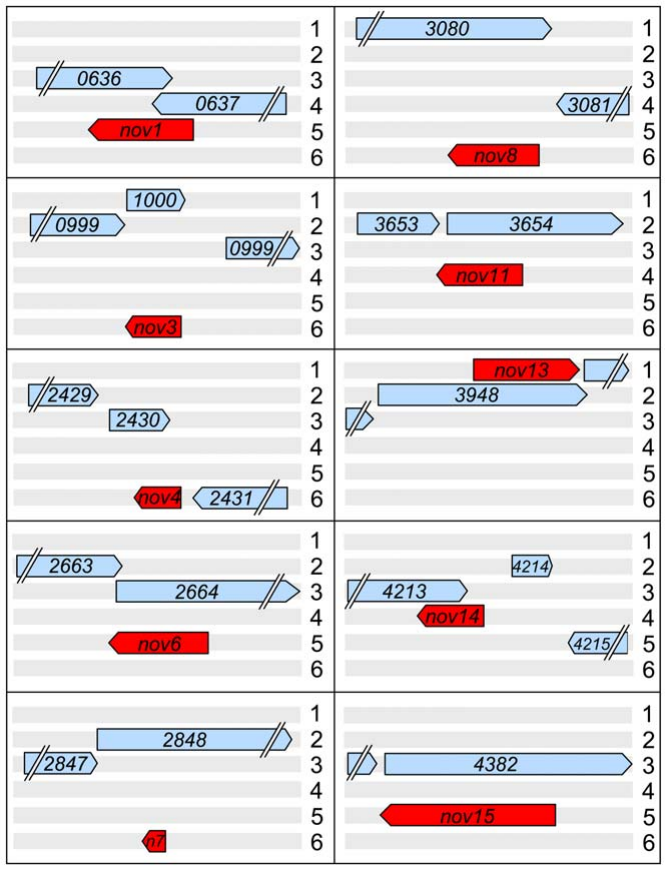
Organization of ten novel ORFs overlapping predicted genes in *P. fluorescens* Pf0-1. The novel ORFs are colored red and indicated by “*nov*” (or *n7* for *nov7*), while predicted Pf0-1 genes are colored blue and labeled with the Pfl01 number of the locus tag corresponding to each in the Pf0-1 GenBank entry. Three forward (numbered 1-3) and three reverse (numbered 4–6) reading frames are shown. Parallel diagonal lines indicate that the complete ORF is not shown to scale. For the length of predicted *nov-*encoded proteins, see Table 1.

An outstanding feature of the *n*ORFs is that ten are predicted to have the bulk of their coding sequences overlapping those of annotated ORFs (Table 1 and Figure 1). Nine are antisense to annotated genes, while one (*nov13*) is encoded on the same DNA strand as an annotated gene, but in a different reading frame. The possibility that the annotated genes were mis-annotated or are pseudogenes and that the *nov* ORFs are the only genes present at these loci seems unlikely since the majority of the annotated ORFs found opposite the *nov* genes are well conserved among *Pseudomonas* spp. or have putative orthologs in other genera (Figure S1). Although overlaps are frequently seen between bacterial genes, these are generally thought to be limited to relatively short lengths. A large-scale analysis of all publicly available annotated microbial genomes revealed that greater than 85% of all identified overlaps were small, sharing less than 30 nucleotides [1]. In contrast, overlapping gene arrangements in which most or even all of one gene overlap another are known to occur in phage and viral genomes [e.g. 19], [20]–[22] and have been verified in the transposable IS5 element [23] and suggested to occur in other IS elements [reviewed in 2]. In both bacterial and viral genomes, the increased density of genetic information that results from overlaps has been suggested to arise from evolutionary pressure toward smaller genomes [24], although it is thought likely that the regulatory implications of overlaps, for example translational coupling, may also be a driving force behind their evolution [1]. Examples of extensive (or complete) overlap between two genes which are antisense to one another in bacteria are rare. These include *hgtA*/*yaaW* in *E. coli* [25], *antE*/*dnaE* in *Bacillus subtilis* [26], *pic*/*set* in *Shigella flexneri* [27], [28], and *cosA*/*Pfl01_0939* in *P. fluorescens* [5]. Direct evidence for proteins produced by both overlapping genes exists only for the latter two examples. The identification of ten *n*ORFs which overlap annotated genes adds considerably to these described examples, and is suggestive of an underestimation of the occurrence of such gene arrangements in bacterial genomes and lack of recognition of the proteins expressed.

To further validate the authenticity of the *n*ORFs and their predicted organization in reference to the previously annotated genes, we carried out reverse transcriptase PCR (RT-PCR) to confirm the production of corresponding mRNA as previously described [29]. This method is capable of discriminating between loci in which one or both strands are transcribed [5]. *P. fluorescens* Pf0-1 was grown under the same conditions from which the peptides were originally isolated (Table 1). RT-PCR products representing each of the *n*ORFs were readily detected. For those *n*ORFs overlapping annotated genes, strand-specific RT-PCR detected expression of both predicted and non-predicted genes, with the exception of *nov13* which overlaps an annotated gene on the same DNA strand (data not shown). The detection of transcriptional activity of the *n*ORFs in addition to the translational activity detected in the proteomic screen substantiates the legitimacy of the *n*ORFs. Since *nov13* is predicted to be encoded within the same strand as the annotated gene, RT-PCR cannot determine whether or not there are two distinct mRNA species. We thus made an attempt to isolate discrete proteins from the two reading frames, as previously described [5], but no protein was detected.

We have previously described several examples of overlapping genes in *P. fluorescens* Pf0-1, where previously unidentified genes (named *iiv*) were found to be encoded antisense to those already annotated [11], [30]. Each of the *iiv* genes was detected by its up-regulation in the soil environment from which Pf0-1 was originally isolated. As expected because of their low basal expression in laboratory culture, we did not observe any peptides which corresponded to the soil induced *iiv* genes (data not shown). Aside from the apparent absence of the native stimuli under our experimental conditions, it is as equally plausible that these genes simply encode regulatory RNA rather than protein [31]. There is, however, experimental evidence supporting that at least one of the *iiv* antisense genes encodes a genuine protein, and furthermore, it appears to contribute to the early stages of soil adaptation [5].

### Are *n*ORFs on Their Way Out or a Potential Source of Innovation?

Although evolutionary selection for overlapping genes in order to increase information density is a plausible explanation for the presence of the overlapping *n*ORFs, as is the case in viral genomes, an alternative explanation is that they have no function. In searches of translated nucleotide databases using TBLASTN, possible orthologs with predicted function were found for only two of the *n*ORFs (Table 3). However, the TBLASTN searches did identify potential (but non-annotated) coding sequences in other genomes with similarity to eight of the overlapping *n*ORF translation products (Table 2 and Figure S2). Half of these are approximately the same size as their counterparts in Pf0-1 (*nov2*, *nov10*, *nov11*, *nov14,* and *nov15*), while the others are up to 50% longer (Table 2). The longer candidates may indicate that in Pf0-1, the wrong start codon has been picked, or that start and/or stop codon mutations have altered the sequences. When sequences similar to other overlapping *n*ORFs were found, the best hits were generally in other *P. fluorescens* strains, with lower similarity hits most frequently in other *Pseudomonas* sp. While the best hits for these eight do not contain stop codons, some lower scoring matches have stop codons interrupting the potential coding sequences. In the case of *nov13*, TBLASTN identified high scoring hits in both *P. fluorescens* SBW25 and Pf-5, but in both cases a stop codon was absent, which would result in a fusion with the downstream gene in the same reading frame. The variation in matches to the *n*ORFs may indicate that most of the *n*ORFs were recently wrought or are on their way to being eliminated from the pseudomonads.

While we know that different growth conditions can trigger the expression of individual *n*ORFs (Table 1), it is possible that their products may not have any biological function. Given the general metabolic cost of amino acid production and cost-based fine tuning of their composition in differentially expressed proteins [32], mutations should accumulate in ORFs to shut down needless energy expenditure if their products do not provide function. There is, however, some indirect experimental evidence suggesting that expression of a functionless protein does not necessarily pose a significant fitness disadvantage if the level of expression remains relatively low [33], [34]. It is thus plausible that expression of a population of such proteins is maintained at low levels (i.e. genetic noise) until a function emerges within the ecological context. Whether selection is acting, or has acted, on the *nov* sequences is unknown. Our BLAST results could be a consequence of selection acting to preserve the already annotated protein on the opposite strand, and the appearance of an ORF that could yield a protein may simply be a side effect of the evolutionary constraints on the annotated gene.

Veloso et al [35] analyzed 315,618 annotated genes in 105 bacterial and archaeal genomes for the presence of alternate ORFs (AlterORF) within the predicted coding sequences. A total of 491,079 AlterORFs were identified. The frequency of these AlterORFs depended to a large degree on the G+C content of a given genome, because of the A+T rich nature of stop codons, and is also influenced by the usage of the leucine codons TTA and CTA, which specify stop codons in an antisense reading frame [35]. Given the high coverage of AlterORFs in G+C rich genomes, including members of the genus *Pseudomonas*, it is possible that some large overlaps may be conserved across genomes. Furthermore, AlterORFs are proposed to provide a framework for novel genetic information to evolve, and are a plausible source of the antisense genes discovered here.

Overprinting is a mechanism that describes the rise of *de novo* proteins from the AlterORFs of existing genes through accumulation of point mutations [36]. In *E. coli,* overprinting has been suggested to be the mechanism behind the evolution of *htgA*, a positive regulator of Sigma-32, which is antisense and completely contained within the gene *yaaW* [25]. Overprinting could be a likely mechanism behind the emergence of the ten *n*ORFs which have considerable overlap with annotated genes in Pf0-1. Overlapping gene organization would provide some degree of protection from genome decay, as long as the function of the protein encoded by the overlapping counterpart is important enough to be maintained through natural selection.

The biological function encoded by each *n*ORF, if any, remains to be elucidated, and further work is required to characterize the functions of the overlapping partners. If we had relied solely on the annotated database as the search field, all the peptides corresponding to the *n*ORFs would have been discarded. There are potentially many more genuine *n*ORFs to be discovered within the larger dataset: some may already have a biological role while others may be on their way to acquiring one or being lost.

## Materials and Methods

### Strains and Culture Conditions

*Pseudomonas fluorescens* strains Pf0-1 [8] and Pf0-2x [10] were routinely grown at 30°C in *Pseudomonas* minimal medium (PMM) [37] supplemented with ampicillin (50 mg/L), solidified where required with 1.5% agar.

### Preparation of Protein Samples

Frozen (−80°C) stocks of *P. fluorescens* Pf0-1 and Pf0-2x were used to start cultures which were grown for 24 h in 2.5 mL of PMM. Each was subcultured 1∶100 into 50mL of rich media {LB (Difco) and King's medium B [38]} and PMM and allowed to grow with shaking. From rich media, cells were harvested at exponential (4.5 h) and stationary (20 h) phases of growth. Cells were harvested from late exponential/early stationary phase cultures in PMM. Cells were harvested by centrifugation (30 min at 5,000×g), the supernatant discarded, and the remaining pellets were flash-frozen in an ethanol/dry-ice bath. The bacterial pellets were stored at −80°C until they were transported on dry-ice to the Pacific Northwest National Laboratory (PNNL; Richland, WA).

At least five independent samples were processed for each condition for each strain and analyzed in-house at the high-throughput proteomic facility of the PNNL. The materials and methods used to prepare the protein samples for mass spectrometry were fully described for the identical set of samples elsewhere [39]: briefly, each sample was lysed, extracted into global, soluble, and insoluble fractions then trypsinized; and further fractionated by ion exchange chromatography.

### Peptide Detection and Analysis

Consistent mass of peptides were analyzed by ultra high pressure reversed-phase HPLC coupled online to a Thermo Finnigan LTQ ion trap mass spectrometer in a data-dependent MS/MS mode. MS/MS spectra were analyzed using SEQUEST [40] against all possible stop-codon to stop-codon open reading frames (ORFs) in the Pf0-1 genome. All identified tryptic and non-tryptic peptides, greater than six amino acids in length, were first filtered by charge state-dependent cross correlation cut-off (Xcorr) scores as described previously [39], and further filtered using a relatively high confidence PeptideProphet [41] cut-off score of 0.8.

### Informatics

New predictions of coding sequences in *P. fluorescens* Pf0-1 were carried out using GenemarkS (http://opal.biology.gatech.edu/GeneMark/genemarks.cgi) and Glimmer (http://www.ncbi.nlm.nih.gov/genomes/MICROBES/glimmer_3.cgi), using default parameters set on the respective websites.

Orthologs of Pf0-1 genes in other *Pseudomonas* genomes were identified by viewing Pf0-1 genes with the GBrowse function on www.pseudomonas.com, with the “putative orthologs” track selected. TBlastN searches of GenBank (NCBI Blast site; http://blast.ncbi.nlm.nih.gov/) were used to identify putative orthologs to annotated and *nov* genes in non-*Pseudomonas* genomes. All multi-sequence alignments were produced using ClustalW2 (http://www.ebi.ac.uk/Tools/clustalw2/index.html) using the ‘full’ alignment option and with default settings for all other options. ORFs for alignment with Nov protein sequences were obtained by analysis of the appropriate *Pseudomonas* genomes using Artemis [42].

### RNA Isolation and RT-PCR

RNA was extracted from *P. fluorescens* Pf0-1 using an RNeasy Mini Kit, and on-column DNaseI digestion (Qiagen). RNA so recovered was treated with RQ1 DNase (37°C, 1 h) (Promega), and subsequently purified using an RNeasy Mini Kit column. First strand synthesis for RT-PCR was carried out using Omniscript (Qiagen) and gene specific primers (Table S1), at 52°C for 1 h. The cDNAs for each *nov* locus were amplified by PCR using the forward and reverse primers listed Table S1. In cases where the *nov* gene was antisense to an annotated gene, the forward primer (Table S1) was used as a gene specific RT primer for the annotated gene to allow confirmation of transcription from both DNA strands. A negative control consisting of a reverse transcriptase-free reaction was used in each experiment. Each RT-PCR product was sequenced at the Tufts University Core Facility (www.tucf.edu). Sequences were aligned with the Pf0-1 genome sequence to confirm the specificity of the reactions.

## Supporting Information

Figure S1 Alignment of genes annotated in P. fluorescens Pf0-1 opposite which are found “nov” genes. Alignments were produced in ClustalW2 (http://www.ebi.ac.uk/Tools/clustalw2/index.html). Where predicted orthologs existed in other Pseudomonas genomes, these were used for alignments. Regions shaded in grey for Pf0-1 lines indicate the portion of the protein specified by DNA which overlaps the nov gene.
(0.17 MB PDF)

Figure S2 Alignment of Nov proteins with putative proteins identified using TBlastN. Alignments were generated with ClustalW2 (http://www.ebi.ac.uk/Tools/clustalw2/index.html ). Full start codon to stop codon sequences in each genome were determined by examining the sequence in the Artemis genome browser (http://www.sanger.ac.uk/Software/Artemis/).
(0.13 MB PDF)

Table S1 Primers used in RT-PCR
(0.06 MB PDF)
