# Supplementary material for: Proteomic Detection of Non-Annotated Protein-Coding Genes in Pseudomonas fluorescens Pf0-1
Source: PLoS One. 2009 Dec 24;4(12):e8455. doi: 10.1371/journal.pone.0008455 (PMC2794547; doi:10.1371/journal.pone.0008455)
Supplement: Figure S1 — Alignment of genes annotated in P. fluorescens Pf0-1 opposite which are found “nov” genes. Alignments were produced in ClustalW2 (http://www.ebi.ac.uk/Tools/clustalw2/index.html). Where predicted orthologs existed in other Pseudomonas genomes, these were used for alignments. Regions shaded in grey for Pf0-1 lines indicate the portion of the protein specified by DNA which overlaps the nov gene. (0.17 MB PDF) [file pone.0008455.s001.pdf]

Figure S1.
Alignment of genes annotated in *P. fluorescens* Pf0-1 opposite which are found "*nov*" genes. Alignments were produced in ClustalW2 (http://www.ebi.ac.uk/Tools/clustalw2/index.html). Where predicted orthologs existed in other *Pseudomonas* genomes, these were used for alignments. Regions shaded in grey for Pf0-1 lines indicate the portion of the protein specified by DNA which overlaps the *nov* gene.

Colors in alignments:
RED = AVFPMILW (small+ hydrophobic (incl.aromatic -Y))
BLUE = DE (Acidic)
MAGENTA = RK (Basic)
GREEN = STYHCNGQ (Hydroxyl + Amine + Basic - Q)

Consensus symbols in the alignments:
* = residues or nucleotides in that column are identical in all sequences in the alignment.
: = conserved substitutions.
. = semi-conserved substitutions.

Pfl01_ = Pf0-1
PA = P. aeruginosa PA01
PA14_ = P. aeruginosa PA14
PFL = P.fluorescens Pf-5
PputW619_ = P. putida W619
PSPPH_ = P. syringae phaseolicola 1448A
PFLU = P. fluorescens SBW25
SELSPUOL_ = Selenomonas sputigena ATCC 35185
BACCELL_ = Bacteroides cellulosilyticus DSM 14838

Pfl01_0636 (3′ end is opposite 3′ end of *nov1*)

```
Pfl01_0636              MS-RHSQCGGPARQRGAIGLMAALTLGMALVFILVVVDSGRLYLERRHLQQIADVAALEA 59
PA4297                  ------MNGWPARQRGAIGILAATTLLLALICLLLVVDTGRLYLEQRNLQRVADVAALES 54
PA14_55820              ------MNGWPARQRGAIGILAATTLLLALICLLLVVDTGRLYLEQRNLQRVADVAALES 54
PFL_0686                MSPLKRFYG-PARQRGAIGLMAAVTFGLALLLMLLVVDSGRLYMEQRKLQRVADNAALEA 59
PputW619_0838           MVPSS-----AARQRGAIGLMAVTTLALALLFMLVVVDSGRLYLEQRKLQRIADMAALEA 55
PSPPH_4415              MSPLDGYTAMPARQRGAIGMMAALTLGLALLCTLIVVDSGRLYLEKRSLQRVADIAALEA 60
                                  .********::*. *: :**:  *:***:****:*:* **::** ****:

Pfl01_0636              ATRGGNCGA--GATANAYAQASVVRNNFPIPSAGRTLAVACGTLNLDASNLRVFAVNA-- 115
PA4297                  ASQGALCGDQSSAQATSFAKASAMLNGFDADAAGSSLSAEVGGVLS-AGGLRSFIASASN 113
PA14_55820              ASQGALCGDQSSAQATSFAKASAMLNGFDADAAGSSLSAEVGGVLS-AGGLRSFIASASN 113
PFL_0686                VSRGGTCQA--GLTAAAYAGQNATRNGFTVAT-GSSLSTSCGSLTTGANGLRTFTANP-- 114
PputW619_0838           AGQSAVCTGN-GPQATAIASVAAARNGHTP---GSPLVASCGYLQTGANSLRTFTSDN-- 109
PSPPH_4415              AGRRGTCSG--AATAPGFATQSATRNGFTPNTGGRTLTTLCGTLTVGLQSQRVFVADS-- 116
                        . : . *    .  * . *   .  *..     * .* .  * :     . * *  .

Pfl01_0636              --ASTEAIRVVVSHTVPQSFAGAIGGLFGGAGRNATINLSATAVAAVPPP-LASLTIRST 172
PA4297                  AAVANEAVHVEVTKSVPGSLVANLGGLFGGG--NANVDLRAEAVARRLPN--ATISAGTG 169
PA14_55820              AAAANEAVHVEVTKSVPGSLVANLGGLFGGG--NANVDLRAEAVARRLPN--ATISAGTG 169
PFL_0686                --AQAVAIRVIATHTVPISVASGVAALFTPGPINLTTQLSATAVAAAPTPTVAQLSIRST 172
PputW619_0838           --ARNEAIRVDVSNTVTTSFAAGIYILTQGGEVPLTSTLQAHAVASKPLPPQAMLSIRTT 167
PSPPH_4415              --TQALAIQVFAAHPVPRSIAAGIVALFEKTPSPANITISATAVAASAAP-LAALTIRSA 173
                          .   *::* .::.*. *... :  *        .  : * ***       * ::  :
```

```
Pfl01_0636         ALSVDTGRSAVLNALFGGLLGGNLSLSAASWNGLVNTNINLLGYLDRLKVDIG---LSAG 229
PA4297             LASVNSGQSALLNPILSGLLGTHIDLSAAAYNGIADAKLSVLDILGADGMGLIGVDTSLG 229
PA14_55820         LASVNSGQSALLNPILSGLLGTHIDLSAAAYNGIADAKLSVLDILGADGMGLIGVDTSLG 229
PFL_0686           LGTVSTAQSSILNPLVGGMLGGSLSLSAVGWNGLLNTNINLLSYLNQLAINLG---VAAG 229
PputW619_0838      LATVDSRQSALLDGLLG-ALGGNVQLGLAGWKGIAATDLNVLNYLDQLAVDLQ---LTVG 223
PSPPH_4415         AVTMDSTRAAILNPLIGSLLGGTLNLSVANWQGLASTDLSLLSYLNRLKTDLN---LTAV 230
                     ::.: ::::*: :..  **  :.*. . ::*:  :.:.:*. *.    .:     :

Pfl01_0636         GYSEVLGNTIAVSQLVQTAINVLDPNGTLSATATILGLQAIKAAAGATQVVLGNLLKVQA 289
PA4297             TIEQLLNTNVGLQQVLAASVNVLAKNGVASVEALRAQLVGVKSAT----LKLGDLLGLSA 285
PA14_55820         TIEQLLNTNVGLQQVLAASVNVLAKNGVASVEALRTQLVGVKSAT----LKLGDLLGLSA 285
PFL_0686           NYTQLLNTTTSVTQLIQAAITVVQANGATADILTALGNLQVAAIN-AAPLKLGDILQMQT 288
PputW619_0838      DYEQLLNADATATQLLEAAVKVLEQSGAAADIVTNLGKVALGAGN-STLLQLGDILDIQN 282
PSPPH_4415         GYSEVLNTSVSVSKLIQSAINVLDPGATLNGTATIAGLQALKLAAGATTVLLGDVLSIQG 290
                      ::*.      ::: :::.*:  ...            :        : **::* :.

Pfl01_0636         GTQ--ATALAVDVRAFDLIQGFVQLANKQNGLVANQTINLG-VAQITASVQVLEPPQLSA 346
PA4297             GEVNSMSQLDTAINALDLIMAVAQVANKNSAVAVNLGIPGLANARLTVVEPKRIVSGPPG 345
PA14_55820         GEVNSMSQLDTAINALDLIMAVAQVANKNSAVAVNLGIPGLANARLTVVEPKRIVSGPPG 345
PFL_0686           GLP--STALDANLQLFQLLQAVIQLSNSNSAVAATLPINVLGLANITVQAKVIEPPQLSA 346
PputW619_0838      GTA--QAGLDASIQLLQLVQGVIQLAASESAATADLPISVLGLINGRVRLKVIEPQQVSA 340
PSPPH_4415         SSD--LAALNTNLRLLDLVQGLAQLANDKTGISTAAQINVGTLAQVNTRFQVVEPPQLSA 348
                   .     : * . :. ::*: .. *:: .:..  .   *      .  .          ..

Pfl01_0636         IGNP-RLAAANP-LGPNRIYVKTAQVRTLLSVNLPLLDTILSLVNAVADLAGPLTNTLNA 404
PA4297             QDES----------GAWRTEVRQSQVRLTADIDSGFLSILATTK---------------- 379
PA14_55820         QDES----------GAWRTEVRQSQVRLTADIDSGFLSILATTK---------------- 379
PFL_0686           IGNPALAKADPM--GANRIYVRTAQVRTLVRVNLSLP-LVSGLSTAVGNLVAPLTPVLNS 403
PputW619_0838      VGDP----------RTDELRVHTAQVRAMISLDLPLLNTVFGLVNAVLDLVSPLTNVLNN 390
PSPPH_4415         IGDPSKIDPLNPKTGANRIYVRTAQMRALVSINLPVLGTITSLANTAGSVVGSLTPILNS 408
                    .:.           .  . *: :*:*    ::  .   :

Pfl01_0636         LLSLNLVGVIDSLTCALGAP-CQTPSIEVLPG------PIRLDVALDVAAANSYVTAFSC 457
PA4297             -----------------------------------------LQLAIEGASGAVWLKSMAC 398
PA14_55820         -----------------------------------------LQLAIEGASGAVWLKSMAC 398
PFL_0686           LLSLNLVATLGSALCLLGAG-CEQLYPAIASS-------SEIDLSLDAGGAIAYVTDYSC 455
PputW619_0838      LLSLNLASTLESVLCLLGVP-CTVTDIVLVPDK------LQLDIGLEVAEATARLDPQSP 443
PSPPH_4415         ALSLNIAGLVTSATCAVGLNSCMVTDFKFLTSDSSASAGPRIDLSLSLASADTYVTGYTC 468
                                                            :::.:. . .   :   :

Pfl01_0636         LTPTN--KTLTTNTTTSVANLKIGRMDPAAVFGSNVTPPAVVVQPLKVIDIGVKTCRRFL 515
PA4297             TRPDT---ALDFGWRSSGATIKIGSPANIDQVGS-----------------------IL 431
PA14_55820         TRPDT---ALDFGWRSSGATIKIGSPANIDQVGS-----------------------IL 431
PFL_0686           PVNNSGTKSLTAHAISSIADLKVGQIDPANAFSSAAEP---VVKPLPLVDLGIRTCYQFL 512
PputW619_0838      DTFRCSPKRLTVQAQSAAAKLAVGRFDSTNAFFTTGTT---AVKALPLIDIGTKRCTKLL 500
PSPPH_4415         TSNTS--KTLSVKTDAGLLSAKVGWIDSTAAFPASTDPSAITALPLPVLDIGTKTCQKIA 526
                            *     :.      :*      . :                        :

Pfl01_0636         VLPVSCDPRVPAVGGGLDISADTTVGQN--ANIPHVYSAP-PAAS-LPEINQPPFYYSYS 571
PA4297             VLGGLVR---------LDLKLDTSIAGS---------------------SGDTRFNVKAD 461
PA14_55820         VLGGLVR---------LDLKLDTSIAGS---------------------SGDTRFNVKAD 461
PFL_0686           VLPGRCDPVVHYAGGGIAVMVNTSVAQN---TQDLVFSSSASPFPIPPNVNQTPTYQTAA 569
PputW619_0838      IL-GTCESRIPYAGGGLGLRVDSKLLGSGPVEHPLVFQG---PVSEPPNIGQPAAYLNMR 556
PSPPH_4415         GLLGNCTARTPFGGGGIGLTFDTVSQSP--LGSSTVVSTT-FSSPNLPEINSAPYFLTSV 583
                    *               : : ::                           ...    .

Pfl01_0636         TG--NVVSSLSD-TLNNLNLNMHGPT-----GSLVSGLGAILSSVKTLLVTTINTVLSPL 623
PA4297             GSDLVQSRRLPESGFLASTLKTSINVNPVVLGLCLPLLCGSTALTDLIVNSVVAPLVDGL 521
PA14_55820         GSDLVQSRRLPESGFLASTLKTSINVNPVVLGLCLPLLCGSTALTDLIVNSVVAPLVDGL 521
PFL_0686           PT-TNIVNSLAG-TLAGINLIVYKPVNNNPLGAIVTGLASAISGVSNLLTPLITGLLSPL 627
PputW619_0838      PVNDNVVGSLSD-TLLGVQLQAFKPTANSGLGDVLVIAGNVLGTVKGIIEPLIRGLLSPL 615
PSPPH_4415         AH--TKPSTLLNGTVSSVKVNVYKPATSNVLGNVITGAAATLNSLTVALDAIVENTLTGL 641
                            *    .    :     .     *  :             :  . :   :  *
```

```
Pfl01_0636         LDTLINTLLMALGINLNQVDVGANLSC-QSGRATLVI 659
PA4297             LTPILNLVLPLLGVQLG--YIDAEILKVDVGRAELLI 556
PA14_55820         LTPILNLVLPLLGVQLG--YIDAEILKVDVGRAELLI 556
PFL_0686           LDPLLNNLLSGLGINLMDVEVGANLTCGQTGKAYLVI 664
PputW619_0838      IDPLVNVLLKVLGIDLVNTDVGANLSC-STGRAQLVL 651
PSPPH_4415         LTSVVDPLFESLGLSLGDADVGANLSC-NIGQATLII 677
                   : .::: ::  **:.*    :.*::   . *:* *::
```

SeqA	Name	Len(aa)	SeqB	Name	Len(aa)	Score
1	Pfl01_0636	659	2	PA4297	556	29
1	Pfl01_0636	659	3	PA14_55820	556	30
1	Pfl01_0636	659	4	PFL_0686	664	48
1	Pfl01_0636	659	5	PputW619_0838	651	42
1	Pfl01_0636	659	6	PSPPH_4415	677	45
2	PA4297	556	3	PA14_55820	556	99
2	PA4297	556	4	PFL_0686	664	30
2	PA4297	556	5	PputW619_0838	651	28
2	PA4297	556	6	PSPPH_4415	677	29
3	PA14_55820	556	4	PFL_0686	664	30
3	PA14_55820	556	5	PputW619_0838	651	28
3	PA14_55820	556	6	PSPPH_4415	677	28
4	PFL_0686	664	5	PputW619_0838	651	46
4	PFL_0686	664	6	PSPPH_4415	677	41
5	PputW619_0838	651	6	PSPPH_4415	677	37

Pfl01_0637 (3′ end is opposite 5′ end of *nov1*)

```
Pfl01_0637          MSSGDKLFDRLLNRQTSTPP----AVMDDPVVPGVGLQLHLDGEGQVIHLTGPLRHVLAQ 56
PA14_55780          --MFEFSRSSSAEAERPEPFSQEGPALWSASLRSWDLCFEMDEQDRVIRVGGRQAYRLQC 58
PA4293              --MFEFSRSSSAEAERPEPFSQEGPALWSASLRSWDLCFEMDEQDRVIRVGGRQAYRLQC 58
PFL_0687            ----------------------------------MGLQLVLDQQGRILSSHGPLRHSLIQ 26
PputW619_0839       ---MSGLFDRWLGSHAAEPA--------AQMPHAVGLQVWLDDQGHVLRLAGPLRTLLAV 49
PSPPH_4416          MPLEKNRLSRWLGRGEAPPVSQAVPVSSEPGPVGVDLYVLLDDEGRIQSISPQLLARLGI 60
                                                       .* . :* :.::          *

Pfl01_0637          QLSAARTPHLLDYLLPHSALAVEGVPADWQGQLLDLDFFSLAGPPLHLRGWVQP--QGDG 114
PA14_55780          AHGLGEQPRPFAEYLERRAPGAPTLAGLRRGERLDLTLRSDAAAPLTCRFQPMQPLDGLG 118
PA4293              AHGLGEQPRPFAEYLERRAPGAPTLAGLRRGERLDLTLRSDAAAPLTCRFQPMQPLDGLG 118
PFL_0687            LPPSREPPHLLAYVLPGSVLALEGGPADWSGQVLDLDLRGAGGQPLHLRGWVQR--CGQE 84
PputW619_0839       P--SHAQARVHDYLYQHSWLALEGVPADWQGQALDLDFQTVHGQALQTRGWLQH--QAIG 105
PSPPH_4416          AAPLSAAQRLMNLLLPNSALVIEGVPHDWLGHMLDLDFKSADAHTLHARGWVQA--HGKG 118
                            :                .    *. *** :    . .*  *        .

Pfl01_0637          WVLQL-LDIGDLLSERRQAHSREQCQWLAAHIRDHLRLCGLARLPQVLGEQLQCLSQRFQ 173
PA14_55780          RSLLLGMDISDLNWQSDSQQHQLQSLSLGKLILSRLRHVSHGHLAEAVQEILESLSGAFQ 178
PA4293              RSLLLGMDISDLNWQSDSQQHQLQSLSLGKLILSRLRHVSHGHLAEAVQEILESLSGAFQ 178
PFL_0687            WHLQL-LDIGDLVQQRQQALGREQCRVFAAQASEQLRLCNLTRLPDVLHDQLTDLARYWH 143
PputW619_0839       WMLQL-FDISDLLRQGAQQEQWLTLRQLAADVGQAVRDCSEDWLTQTTAEQLMRLAEHWH 164
PSPPH_4416          WLLQL-LDISDLMEEASAARSRQQCLRFAGQMAERVRVCNVDRLTAVVTEQLEELAQLWR 177
                      * * :**.**  :            :.    . :*  .   *. .  : *  *:  ::

Pfl01_0637          VPCLAVALLDEETEGWQIHQHYSAPDAPRLWDMAQRLGTGLDSLNGAAPQRVLP----AE 229
PA14_55780          MQAIALLLGDGKGFCTVFASHVRPGSDSLLRPTLQLADDDLREGAGARLLRRGE-----G 233
PA4293              MQAIALLLGDGKGFCTVFASHVRPGSDSLLRPPLQLADDDLREGAGARLLRRGE-----G 233
PFL_0687            IPCLGLALLDEQEQGWVIHSCYSAHDAPRLWQEGQALGAGLDSLSGNTPQHLNQIQGLNE 203
PputW619_0839       AGSVRLMLADQS--GWRAHTCS---ESDWPWPDDQRLQPWLQALPAHFQQVAP------D 213
PSPPH_4416          VPCLALVLPEVSGSGWRVYCQYSAHTAPELWQVGQRLGNALDRFDANITHQLRFGGGVDQ 237
                      .: : * : .                      *     *    .

Pfl01_0637          HPRLSSVFGPAEGFAVPYRDAHGVAAWLLFGCFSAQPHTAELSDHDWLQLAAAVAAPLLE 289
PA14_55780          ASTLLRQIGEDALYLVPAAMRGGRLGALLVRPMSLEQLAQGPAPQDWQYLAELLANQVAD 293
PA4293              ASTLLRQIGEDALYLVPATMRGGRLGALLVRPMSLEQLAQGPAPQDWQYLAELLANQVAD 293
PFL_0687            HPRLQGIFGHREGFLVPYRDDHGVAAWLLCGCYAVQQHAPYLLDRDWLMLAAALAGPLLS 263
PputW619_0839       LPQWRATCAGLPLPFLVPYSHGHEVQAWLLCAGAPSVP-----SPEAAMALLQALLEPLLA 268
PSPPH_4416          GP-LQSAFGNADGFLIPHSRDNVAKAWLMFGFYNPQQQAPDLGEREWLNLCSALAGPVLF 296
                     .      .    : :*        . *:               .    *   :   :

Pfl01_0637          RLREHRHHLQLERLEILQSLLGTGWWEVFSDSAEVLLADALAVSLGLSAG--RLPIQAWF 347
PA14_55780          RCELHEQHDSSRKLGLLQEMIGGGWWRYWAEQELFELAPALHDSLGLTGEYRRVPLEHLQ 353
PA4293              RCELHEQHDSSRKLGLLQEMIGGGWWRYWAEQELFELAPALHDSLGLTGEYRRVPLEHLQ 353
PFL_0687            RLREHRHHLHLERLEALQGLLGAGWWELCPDTAEILLASRLAASLHLPEDGARLNVQAWL 323
PputW619_0839       RLRQHQLQQRSRHLDELQQQLGAGWWQ-WPVQGPLHFDPALAASLDLPSA---VSIEQWL 324
PSPPH_4416          RLSEQHNRHHVERLAVLQDLLGTGWWELMPARDEILLAPQLARSLRLGDRVESLSRQDWL 356
                    *   :. :   .:*  **  :* ***.  .    . :   *  ** *      :  :

Pfl01_0637          ARVHPADREELRSRLQALQDEGQALELCVRLQPVDQAQPQ-WFRLQGQSLGIGAERRLVG 406
PA14_55780          GLLQPADADELGLRLRASLRSGQALAQDLCLRQPDSCGERRWLRIEGRPLGRGSALGLSG 413
PA4293              GLLQPADADELGLRLRASLRSGQALAQDLCLRQPDSRGERRWLRIEGRPLGRGSALGLSG 413
PFL_0687            ELIHPADRGELSSRLQELK-LGKPLLLNVRLHEPDPSAPQRWYRLQGQALGRGESQRLVG 382
PputW619_0839       ARLHPADREAAQMALEQLR-NGVALSLNVRLLEADRGAEPRWLHWVGQLHG-GQAH---G 379
PSPPH_4416          ALIHPADRQELSSRLAQLRDKGTPLLLCVRLAQSDPAQETLWYRIQGQALISGQVQRVLG 416
                      ::***       *      * .*   : *   *      * :  *:    *      *

Pfl01_0637          FMLDISDIKNQQQQAAAAHARLDNLIASAPAVIYVQRYVEGALQPTFFSASLQPLLGWSL 466
PA14_55780          VLLDISEGRRQEERAQAAHARLRSLIDSAPVVIYVQRVEQGHLVPEFYSESASNLLGLDL 473
PA4293              VLLDISEGRRQEERAQAAHARLRSLIDSAPVVIYVQRVEQGHLVPEFYSESASNLLGLDL 473
PFL_0687            FMLDISDIKNQQQQAAAAHARLDNLIASSPAVIYVQRYQEGALQPSFFSASLQPLLGWSL 442
PputW619_0839       FVLDISALKAQELQASAARARLENLIASSPAVIYIQRCCEGALHGEFFSASLTPMLGWPK 439
PSPPH_4416          FMLDVSDIKNQETEAAAAHARLDNLIASSPAVIYVQRYDHGNLEPTFFSDSLTPLLGWTL 476
                    .:**:*  : *: .* **:*** .** *:*.***:**  .* *   *:* *   :**
```

```
Pfl01_0637          ADCADGALVNHVHPDDRAAYFERTRQLLREGSVSTRYRMLDTQGNTHWLLDEAKLLRDDL 526
PA14_55780          QGQSWQALAERVHPDDLEAFFARGRELLREGRVKTRYRLADGQGNWHWLYDEAKLLRDAQ 533
PA4293              QGQSWQALAERVHPDDLEAFFARGRELLREGRVKTRYRLADGQGNWHWLYDEAKLLRDAQ 533
PFL_0687            ADCTAQPLAEWVHPDDRDLYFQRCRQLLREGSVHTRYRLRDRHGVYHWVLDEAKLLRDDL 502
PputW619_0839       DSELARQPGQAVHPHDRALWLERTRTLLRDGQVRSRYRLRDHLGGYHWVLDEARLLRDDL 499
PSPPH_4416          ADCVDGQLAEYIHPEDRDIWFERNRQLLRGGVVSRRYRLRNKNGHYHWLLDEARLLRDDL 536
                     .       : :**.*   :: * * *** * *  ***: :  *  **: ***:****

Pfl01_0637          GLPVEAVGLWLDVTDATLAAERIKTSEERYRILVEDSPAMICRYRPDLTLTFGNRPLAMY 586
PA14_55780          GLPSEAVGLWLDVTEQHLAAQRIAESEERYRVLVEDSPALICRYTADLVLTYVNRTFADS 593
PA4293              GLPSEAVGLWLDVTEQHLAAQRIAESEERYRVLVEDSPALICRYTADLVLTYVNRTFADS 593
PFL_0687            GLPVEAVGLWLDVTEATLAAEQVRQSEERYRILVEDSPAMICRYRPDLTLTFGNRPLAAY 562
PputW619_0839       GQPVEVVGLWLDVSEATEAAERVRQSEERYRVLVEDSPAMICRYTPDLQLLFGNRPLAEQ 559
PSPPH_4416          GMPVEAVGLWLDVTEATLAAEHVRQSEERYRILVEDSPAMICRYSPDLVLSFGNRPLANY 596
                    * * *.*******::   **:::  ******:*******:**** .** * : **.:*

Pfl01_0637          LECAPEQLPGVNLGSWMSDEQREAFVQRLAQLTPDAPVSSAEINLRLPGREHAWWIWSDR 646
PA14_55780          LATSPERLVGRRLDEWLAAEDASALRARLLGSPREGASEVPELRFNLPGQRFLWLVWAER 653
PA4293              LATSPERLVGRRLDEWLAAEDASALRARLLGSPREGASEVPELRFNLPGQRFLWLVWAER 653
PFL_0687            LECAPEELPGSNLGQWLSAEQREGFERRLKLLSPEYPVSTAEINLQLPGREHAWWVWSDR 622
PputW619_0839       LECTPEQLSEMNLGQWMSDGQRLSFIQRLAALTPEQPLGSAEICLQLPGHRHAWWVWAER 619
PSPPH_4416          MECTPEQLTGINLGDWLSDEQREAFIRRIGQLTPQAPVSTEEICIELPGREYAWWVWADR 656
                    :  :**.*   .*..*::  :  .:  *:   . : .    *: :.***:.. * :*::*

Pfl01_0637          GVFDEQGRLLEVQAVGRDNTEVRRSQQQLTQSAKMATLGEMATGLAHEINQPLNVMRMAI 706
PA14_55780          PLFDARGELCEVQAVGRDNTPVRRAQQQLAQGAKMASLGEMVSGLAHEVKQPLHVLRMTL 713
PA4293              PLFDARGELCEVQAVGRDNTPVRRAQQQLAQGAKMASLGEMVSGLAHEVKQPLHVLRMTL 713
PFL_0687            GVFDEQGQLLEIQAVGRDNTEVRRAQQQLTQSAKMATLGEMATGLAHEINQPLNVMRMAI 682
PputW619_0839       GLFNEQGQLLEVQAVGRDNTQVRQSQQQLMQGAKMATLGELATGLVHEINQPLNVMRLAI 679
PSPPH_4416          GIFDEHGTLVEVQAVGRDNTDVRRSRMQLNQSAKMATLGEMSTGLAHEINQPLNVMRMAV 716
                     :*: :* * *:******** **::: ** *.****:***: :**.**::***:*:*:::

Pfl01_0637          VNVLKRLGNGDVQIDYLTDKLNRIDTQVQRAARVVDHMRVFGRRSEIEQHPFNPLDAIEG 766
PA14_55780          FNMRQRMNSVGLDGDYLGEKLERMDAQVLRVDRLVSHLGVFSRKSALEALPFDPYAAFEG 773
PA4293              FNMRQRMNSVGLDGDYLGEKLERMDAQVLRVDRLVSHLGVFSRKSALEALPFDPYAAFEG 773
PFL_0687            VNVLKRLGNGDVQIDYLTDKLKRIDAQVQRAARVVDHMRVFGRRSEVEQQPFNPAHAIEG 742
PputW619_0839       ANTLKRVESGVVEVEYLTGKLQRIDAQVGRVARLVEHMRTYGRRSALEHQVFAAWTAVDG 739
PSPPH_4416          VNVLKRLGRGDVDIEYLTEKLNRIDTQVQRAARVVDHMRVFGRRSEVEEHLFDPAQAVEV 776
                     *  :*:    :: :**  **:*:*:** *. *:*.*: .:.*:* :*   * .  *.:

Pfl01_0637          TLSLLSEGLRGKGVELRIGEANFQLQVRGYVDQLEQVLINLMVNARDALLSKRESNRDFQ 826
PA14_55780          ALGLLGEGLRQHAIEVECPAPTQRMVVRGQADQLEQVIINLLANARDALLGNPGLASR-R 832
PA4293              ALGLLGEGLRQHAIEVECPAPTQRMVVRGQADQLEQVIINLLANARDALLGNPGLASR-R 832
PFL_0687            TLSLLAEGMRGKGLELRISELGFEVQVLGFVDQLEQVLINLLVNARDALLGKQEKQHDFK 802
PputW619_0839       AVALLAEGLRGKGVALQVEEPDFCPEVQGHEDQLEQVLINLMVNARDALLERQVAS---- 795
PSPPH_4416          TLSMLAEGMKGKGVQLRVGTMIEDVQVRGHVDQLEQVLINLMVNARDALLSRREKDRDFE 836
                    ::.:*.**:: :.: :.         * *  ******:***:.******* .

Pfl01_0637          PWIAVHAEHDEHVVRLWVEDNGGGIDPRLLERIFEPFFTTKPVGVGTGLGLSVSYGIVEN 886
PA14_55780          VRLEQVACREPGWVELHVHDNGGGIEPLLLERIFEPFFTTKAEGKGTGLGLSVSHDLVRN 892
PA4293              VRLEQVACREPGWVELHVHDNGGGIEPLLLERIFEPFFTTKAEGKGTGLGLSVSHDLVRN 892
PFL_0687            PWIAVYGQRDEQWVRLWVEDNGGGIDPRLLERIFEPFFTTKPVGIGTGLGLSVSYGIVQN 862
PputW619_0839       PRIGICQRIEQGQLCLQVEDNAGGIDPCLIERIFEPFFTTKPAGVGTGLGLSVSFGIVEA 855
PSPPH_4416          PWISVEAERDENIIRLAVQDNGGGIDPRLLERIFEPFFTTKPVGVGTGLGLSVSYGIVDQ 896
                      :      :   : * *.**.***:* *:***********. * *********..:*

Pfl01_0637          MGGRLSVRNGDDGARFCIELPIALDDQITSVARPD 921
PA14_55780          MGGSLTAANQGEGALFVVRLPLAAPAEAGG----- 922
PA4293              MGGSLTAANQGEGALFVVRLPLAAPAEAGG----- 922
PFL_0687            MGGRLSVSNSQDGARFCIELPIAEADQITR----- 892
PputW619_0839       MGGRLEALNGVDGACFRVLLPIYNTN--------- 881
PSPPH_4416          MGGQLSVGNVGEGARFQIELPIFNAG--------- 922
                    *** * . *  :** * : **:
```

SeqA	Name	Len(aa)	SeqB	Name	Len(aa)	Score
1	Pfl01_0637	921	2	PA14_55780	922	40
1	Pfl01_0637	921	3	PA4293	922	40
1	Pfl01_0637	921	4	PFL_0687	892	72
1	Pfl01_0637	921	5	PputW619_0839	881	52
1	Pfl01_0637	921	6	PSPPH_4416	922	60
2	PA14_55780	922	3	PA4293	922	99
2	PA14_55780	922	4	PFL_0687	892	41
2	PA14_55780	922	5	PputW619_0839	881	35
2	PA14_55780	922	6	PSPPH_4416	922	38
3	PA4293	922	4	PFL_0687	892	41
3	PA4293	922	5	PputW619_0839	881	36
3	PA4293	922	6	PSPPH_4416	922	38
4	PFL_0687	892	5	PputW619_0839	881	52
4	PFL_0687	892	6	PSPPH_4416	922	63
5	PputW619_0839	881	6	PSPPH_4416	922	50

Pfl01_1000 (overlaps *nov3*)

```
Pfl01_1000          MYKLAFFVPDSHVEVVKEAVFAAGGGRIGDYDHCAWQVLGSGQFRPLDGSQPFIGQAGQV 60
PA14_15770          MYKLCFYVPESHLDVVKQAVFAAGGGRIGAYDSCCWQSLGQGQFRPLDGSQPYLGQVGQV 60
PA3762              MYKLCFYVPESHLDVVKQAVFAAGGGRIGAYDSCCWQSLGQGQFRPLDGSQPYLGQVGQV 60
PFL_1077            MYKLSFFVPPSHVESVKSAVFAAGGGRIGAYDQCAWQVLGQGQFRPLDGSQPFLGQTGQV 60
PputW619_4188       MYKLAFFVPASHVDVVKAAVFAAGGGRIGGYDHCAWQTLGHGQFRPLDGSQPFLGQTGQV 60
PSPPH_1342          MYKLAFFVPPSHVDEVKSAVFAAGAGRIGAYDHCSWQVLGQGQFRPLNGSQPFVGQSGVV 60
                    ****.*:** **:: ** ******.**** ** *.** ** ******:****::** * *

Pfl01_1000          ERVEEWKVELVVGDELIRSVVAALKLSHPYETPAYEVWRLEDF-- 103
PA14_15770          EHVAEWKVELVVADELIHASVKALKAAHPYETPAYEVWRLTDMVF 105
PA3762              EHVAEWKVELVVADELIHASVKALKAAHPYETPAYEVWRLTDMVF 105
PFL_1077            ERVEEWKVELVVADELIRDLVLALKQSHPYETPAYEVWRLEAF-- 103
PputW619_4188       EVVEEWKVELVVADDLIAQVVAALRQSHPYETPAYEVWRLADF-- 103
PSPPH_1342          ESVEEWKVELVVADDLIQQAVLALKQSHPYETPAYEVWRLEEF—103

                    * * ********.*:**   * **: :*************  :
```

SeqA	Name	Len(aa)	SeqB	Name	Len(aa)	Score
1	Pfl01_1000	103	2	PA14_15770	105	75
1	Pfl01_1000	103	3	PA3762	105	75
1	Pfl01_1000	103	4	PFL_1077	103	85
1	Pfl01_1000	103	5	PputW619_4188	103	84
1	Pfl01_1000	103	6	PSPPH_1342	103	79
2	PA14_15770	105	3	PA3762	105	100
2	PA14_15770	105	4	PFL_1077	103	77
2	PA14_15770	105	5	PputW619_4188	103	76
2	PA14_15770	105	6	PSPPH_1342	103	73
3	PA3762	105	4	PFL_1077	103	77
3	PA3762	105	5	PputW619_4188	103	76
3	PA3762	105	6	PSPPH_1342	103	73
4	PFL_1077	103	5	PputW619_4188	103	82
4	PFL_1077	103	6	PSPPH_1342	103	84
5	PputW619_4188	103	6	PSPPH_1342	103	81

Pfl01_2430 (overlaps *nov4*)

No predicted orthologs in *Pseudomonas* genomes; no matches in GenBank.

Pfl01_2664 (overlaps *nov6*)

No predicted orthologs in *Pseudomonas* genomes; there are matches in GenBank.

```
Pfl01_2664          MEQFKQFNASKTEVIKSLDNLQKLVGALEALGLDVSQDIEKIRKAIGDVQSDALRIALLG 60
Shewana3_2033       MEQLKQFEVEKQSAISTLQQLKEVLTELGAIGMDVSVDIQKLEAALNTIKDDVLRIALLG 60
E. coli LeoA        MEQFKQFSIEKQAAINSLLQLRGMLEMLGEMGINISDDLQKVTSAINAIESDVLRIALLG 60
SELSPUOL_01925      MEKLERLSDWEACAKRRYLAVQEKMEELHDLNVDCAEDLQKIEDKLGSMGQEKISLALVG 60
BACCELL_05373       METSKNSGNEKQ---HRLIALKSLVEKGITLGLDLTDVLEKIENVIKSSKDNIIRMVLLG 57
                    **  :.    :         ::  :     :.:: :  ::*:   :    .: : :.*:*

Pfl01_2664          AFSDGKTSVVAGWLGQVMKDMKIDTNESSDALAIYRPDNLQDRCEIVDTPGLFGDKEKAA 120
Shewana3_2033       AFSDGKTSVIAAWLGKIMADMKIDMDESSDRLAIYRPEGLPDKCEIVDTPGLFGDKQKED 120
E. coli LeoA        AFSDGKTSVIAAWLGKVMDDMNISMDESSDRLSIYKPEGLPDQCEIVDTPGLFGDKEREV 120
SELSPUOL_01925      AFSDGKTSVIAGLFGEELEGMQISVDESTDEIQIYEPNNPKISCRIVDTPGLFGTKEKQR 120
BACCELL_05373       TFSDGKTSVVAGLLGKLEDSMKIDQDESSDELKVYRPNGLKEGFEIVDTPGLFGTKEKEV 117
                    :********:*. :*:   .*:*. :**:* : :*.*:.     .********* *::  

Pfl01_2664          ENGALVQYGDITKNYISEAHLIFYVVDATNPLKESHQDIVRWVLRDLNKLSSTIFVINKM 180
Shewana3_2033       EN-LQIMYEDITRNYISEAHLILYVVDATNPLKESHHQIVRWVLRDLNKLSSTVFIINKM 179
E. coli LeoA        DG-RLVMYEDLTRRYISEAHLIFYVVDATNPLKESHSDIVKWVLRDLNKLSSTIFVINKM 179
SELSPUOL_01925      SD-GVVQLGEIAKRYLAEAHMLLYVVESKNPIKDSHKDTLRWIMKDLQKTDRMIFVINKM 179
BACCELL_05373       DG-KNIRYSELTQKYISEAHVVIYVCDAVTPLKESHKDIIKLVMRDFNKLESTIFVINKM 176
                    ..   :   :::::.*::***:::** :: .*:*:** : :: :::*::*  .  :*:****

Pfl01_2664          DEV-ADLRDAEEFEQQSTIKKNNLLDKLQRFVDLSAAERDAINVVCVASNPNSRGLEFWL 239
Shewana3_2033       DEV-TDLSDPTLFSEQAAIKTETLKEKLQRAASLTDEEVKALRIVCIASNPNGRGLPFWF 238
E. coli LeoA        DEV-TSLTDQALFDEQAAIKKANLKGKLQRAADLTAQECEQLNIVCVASNPNGRGLTYWF 238
SELSPUOL_01925      DDV-ADLSDEEEFLSQAAIKKEAVREKLRELVGVRADDAAKVRIVCIAADPDRRGMDFWK 238
BACCELL_05373       DEAGTDMLDEEDYNEMSSIKKDNLLKRLRDTIGLTAEEESKMRIACVAADPKGKGLKHWF 236
                    *:. :.: *   : . ::**.  :  :*:   .:   :   :.:.*:*::*. :*: .* 

Pfl01_2664          EQKDLYDQRSRISELKVVTQQVLDGSGRETLIKKTGIDVVRDVLGKKIALAQDELIKFRL 299
Shewana3_2033       EKLDVYRNRSRIDELKKTTTSILDSSVRSTIIAKTGIDVVAGVVSQRVLAAQNSLDELEV 298
E. coli LeoA        TKPEHYESRSRINDLKNAATEILKTNVPEVLLVKTGMDVVKDIVIQRVTLASRHLDELNT 298
SELSPUOL_01925      EHTDVYEKRSHIVDLEDAIEGVLQKHSAEEFFANAALGVCKAVLGRYIEEVPTFVKAQEK 298
BACCELL_05373       TDMDGYLKRSHIEVLREYISSITDTLDTDAAKNNAKTAVIKDVLLSMSKRVDAVFGPLSL 296
                     . : * .**:*  *.     : .    .    ::   *   ::      .   .     

Pfl01_2664          LADNQRRDSARIQEDIAEGKRKVLATVGNLYEELNNLETDLLNSIRTLPRDQVRAFLESQ 359
Shewana3_2033       FAKQREQECERIREDLNNGKREVKRLAGQLFNELEGLEKQLMGKLRPLDFEDIRPFMEDE 358
E. coli LeoA        FVEKNDEDMHRFSNDIKQSRIEVKRLAGELFEELNLMEKQLMSQLRPLDLDDIRPFMDDE 358
SELSPUOL_01925      MREKLAESVSRQEADFQETRKHLLLAGEECWEELAAYRQRLLDGIHALSLESAEKFIDLE 358
BACCELL_05373       ALRRYDDELKDMNNDLNILKGELNQSKAIMTERLNDLKKSIKMDINNATQNSFGTVIDDD 356
                       .   .      *:   : .:        :.*   .  :   :.    :.   .:: :

Pfl01_2664          IGFSNNDIG-EKLRFRIDHLFSTAFNQSSSIMSGISAKIEHQLDSSSDFADSITASALSA 418
Shewana3_2033       IGYTQDNIG-YKLSIKIKTIVDSYYDQTSLITSRLNQEINSQLDSSTSFLDSLGSDALKF 417
E. coli LeoA        LGYTEDGVG-FKLHLRIKQSVDRFFEQSTAVSQRLSDDITRQLSSSESFLSGLGEGAFRS 417
SELSPUOL_01925      FGRSGDTIG-KHFEQQLTHIMEKHLGRMREDIETFKKKFEQETDVQSNIFLGLGRQAQSW 417
BACCELL_05373       LGMEDKKVTGYILLRKVNQILSECAEKNNATLSMKEIDFNLRFDKQDSMLKDVLKQGGSY 416
                    :*   . :    :  ::   ..    :     .  . .:  . . . .:  .:   .   

Pfl01_2664          SSRALTAVSKIPVESIKSGVFLARETLKNLTGYVYKFKPWEATKLAANISRWAGPIGAGI 478
Shewana3_2033       VGSGLSNVSKISPDAIKATIFVARDLLAKTTGVVYKFKPWQASKLAAGISKWAGPAGMVF 477
E. coli LeoA        LGGAFKGVSKISPATLKTTILAARDTIGKLTGYVYKFKPWEATKLAGSIAKWAGPVGAAF 477
SELSPUOL_01925      IKYIP---PTKMVDFARKGIFIGRDLLKRIG-IIIKFKPWQVVKFAKAVPILT----VLL 469
BACCELL_05373       LG---------KVNISGEMVKAARDIFAKS----YKFKPWGAVNLGKSMTKLAKVGGAAL 463
                             :  .*: : .      ***** . ::.  :.  :      :
```

```
Pfl01_2664         QVLTDVISVAQQQRAETELKQLQGDLAEVVKSYFAEPMKLLMDHEKVMDIFAPQIKAFEK 538
Shewana3_2033      SLTADFYNAYKASELEAELKETKNEISTMIKEAFKDLYDIVSDDQKLFDFFAPQLKIFDQ 537
E. coli LeoA       TIGSDLWDAYKAHEREQELKEVKASLAKIIKEPFEDIYDVLSSDEKMFAFFAPQIQQMEQ 537
SELSPUOL_01925     ELLMLVVDALVQSKRDARLKELKETLLQGVEETFQEIQSTLQG-DALFENYVPDFLSVQS 528
BACCELL_05373      ALAIEVVSFISAARNEKKLKDLKADIVGEVDKIFKSIFEMFDKDDKYYENFASSYIEMTK 523
                    :   . .     . : .**: :  :   :.. * .  . .   :     :...   . .

Pfl01_2664         ILVEQEASLDDLRNRQTQLAELETHLSQAFDSQNVIEGEYGVV 581
Shewana3_2033      LITDTESQVKDIRVSQNSLRGISEKLKSLPSY----------- 569
E. coli LeoA       VVTELAEKSQAIRDNRQKLSLIQTQLAQLMVPATENHTAR--- 577
SELSPUOL_01925     DLEAAQKELQDLKENMRLLAVWTEAAKELQLGLENLR------ 565
BACCELL_05373      RLHARMQEISVMRHKVANLEDYGRSLKDWFKEDAEDADFEEI- 565
                    :     . . ::     *        .

SeqA Name            Len(aa)  SeqB Name            Len(aa)  Score
=================================================================
1    Pfl01_2664      581      2    Shewana3_2033   569      48
1    Pfl01_2664      581      3    E.              577      45
1    Pfl01_2664      581      4    Selenomonas     565      30
1    Pfl01_2664      581      5    Bacteroides     565      30
2    Shewana3_2033   569      3    E.              577      57
2    Shewana3_2033   569      4    Selenomonas     565      29
2    Shewana3_2033   569      5    Bacteroides     565      31
3    E.              577      4    Selenomonas     565      28
3    E.              577      5    Bacteroides     565      32
4    Selenomonas     565      5    Bacteroides     565      27
=================================================================
```

Pfl01_2848 (overlaps *nov7*)

```
Pfl01_2848      MKITVFGSGYVGLVQAAVLAEVGHDVVCMDIDEKKIELLRQGHVSIFEPGLASLVREGLD 60
PA14_18300      MKISVFGSGYVGLVQAAVLAEVGHDVLCMDIDRNKVERLAQGLASIYEPGLDALLREGLD 60
PA3559          MKISVFGSGYVGLVQAAVLAEVGHDVLCMDIDRNKVERLAQGLASIYEPGLDALLREGLD 60
PFL_3050        MKISVFGSGYVGLVQAAVLAEVGHDVICMDIDEQKVKQLQQGHVSIFEPGLASLVKENLE 60
PSPPH_2809      MRISVFGSGYVGLVQATVLAEVGHDVVCMDIDQAKIDQLRQGQVHIYEPGLANLVRDNLD 60
                *:*:************:*********:*****. *:. * ** . *:****  *:::.*:

Pfl01_2848      AGRLQFTCDEILAVQHGKVAFIAVGTPSNEDGSADLRYVLSVGDAVARHREKPLILVEKS 120
PA14_18300      SGRLRFSSDARLAVEHGRVQFIAVGTPPGEDGAADLGAVFAVADAIAEHRRKPVIVVEKS 120
PA3559          SGRLRFSSDARLAVEHGRVQFIAVGTPPGEDGAADLGAVFVVADAIAEHRRKPVIVVEKS 120
PFL_3050        AKRLHFTHDEKLAVQHGQVLFIAVGTPSSEDGSADLRYVLSVGDAVARHREQPVILVEKS 120
PSPPH_2809      HERLHFTTDERLAVEHAEVLFIAVGTPSRADGSADLGGFFAVGEAIARHRCEPLIIVEKS 120
                  **:*: *  ***:*..* *******.  **:***  .: *.:*:*.** :*:*:****

Pfl01_2848      TVPVGTGDTLRAHLDKALVKAGRLLQFDIVSNPEFLKEGSAVADCRRPDRIVIGCEREEV 180
PA14_18300      TVPVGTGDRLRAHIERRLEADGRELEFEIVSNPEFLKEGSAVADCRRPDRIVVGCDNEEV 180
PA3559          TVPVGTGDRLRAHIERRLEADGRELEFEIVSNPEFLKEGSAVADCRRPDRIVVGCDNEEV 180
PFL_3050        TVPVGTGDTLKAHIEKVLD--GRALQFDIVSNPEFLKEGSAVTDCRRPDRIIIGCEREEV 178
PSPPH_2809      TVPVGSGDALRGHIDKVLRQAGRLLQFDIVSNPEFLKEGSAVNDCRRPDRIVIGCENEAV 180
                *****:** *:.*::: *   ** *:*:************** ********::**:.* *

Pfl01_2848      RDVMRDLYSPFNRNHDRIMFMDLRSAELTKYAANCMLATKISFINQIAELAEHLGADIES 240
PA14_18300      RQVMRELYEPFNRNHDRMLFMGLRSAELTKYAANGMLATKISFINQIAELAEHLGADIEA 240
PA3559          RQVMRELYEPFNRNHDRMLFMGLRSAELTKYAANGMLATKISFINQIAELAEHLGADIEA 240
PFL_3050        RDVMRDLYAPFNRNHDRIMFMDLRSAELTKYAANCMLATKISFINQIAELAEHLGADIEA 238
PSPPH_2809      RKVMRELYAPFNRNHDRIMFMDLRSAELTKYAANCMLATKISFINQIAELAEHLGADVES 240
                *.***:** ********::**.************ **********************:*:

Pfl01_2848      VRQGIGADSRIGYHFIYPGCGYGGSCFPKDMRALIHSAEEAHCSSDLLQAVEAINQRQKH 300
PA14_18300      VRQGIGADPRIGYHFIYPGCGYGGSCFPKDMRALIHSAEQAHCSSDLLQAVEAINRRQKH 300
PA3559          VRQGIGADPRIGYHFIYPGCGYGGSCFPKDMRALIHSAEQAHCSSDLLQAVEAINRRQKH 300
PFL_3050        VRLGIGADTRIGYHFIYPGCGYGGSCFPKDMRALIHSAQQAHCSSDLLQAVEAINERQKH 298
PSPPH_2809      VRLGIGADSRIGYDFIYPGCGYGGSCFGKDIRALIHSAREAHCSNDLLSVVEAINERQKN 300
                ** *****.****.************* **:*******.:****.***..*****.***:

Pfl01_2848      KLFERINAFYKGELRGKTFAVWGLAFKPNTDDMRDAPSRVLLESLWAAGANVRAFDPEAM 360
PA14_18300      KLFERIRVFYDGDLRGKTFALWGLAFKPNTDDMRDAPSRELMEALWRHGAQVRAYDPEAM 360
PA3559          KLFERIRVFYDGDLRGKTFALWGLAFKPNTDDMRDAPSRELMEALWRHGAQVRAYDPEAM 360
PFL_3050        KLFERIKAFYKGDLKGKTFALWGLAFKPNTDDMRDAPSRVLLDALFAAGAQVRAFDPEAM 358
PSPPH_2809      KLFERVNAFYQGDLRGKTFALWGLAFKPNTDDMRDAPSRMLMEALWEAGASVRAFDPEAM 360
                *****:..**.*:*:*****:****************** *:::*:  **.***:*****

Pfl01_2848      QETQNLYPDESKLMLMGTPESVLAGSDALIICTEWQQFKAPDFDLIQQRLKAPVIFDGRN 420
PA14_18300      QETQRLYGHDERLSLMGTPEATLGGADALVICTEWQQFKAPDFELLKERLKAPVIFDGRN 420
PA3559          QETQRLYGHDERLSLMGTPEATLGGADALVICTEWQQFKAPDFELLKERLKAPVIFDGRN 420
PFL_3050        QETQRLYPDESRLMLMGTPESVLVGADALVICTEWQQFKAPDFDLIQQRLKAPVIFDGRN 418
PSPPH_2809      QETQRIYGHRKDLILMGTPESVLNDADALIICTEWQQFKAPDFELIQQRLNAPIIFDGRN 420
                ****.:* . . * ******:.* .:***:*************:*:::**:**:******

Pfl01_2848      LYDAERLARNGFHYFPMGRGESRTLPIPLQQWPHASDVA----- 459
PA14_18300      LYDPERMARHGFHYYPMGRGQSCSLPINEASLAQEDGVRLLRQA 464
PA3559          LYDPERMARHGFHYYPMGRGQSCSLPINEASLAQEDGMRLLRQA 464
PFL_3050        LYDAERLARNGFTYFPIGRGESRNLPIPHQQWSAEA-------- 454
PSPPH_2809      LYDTERLATRGFHYFPIGRGESCDLPIPQKRWTPYDQLTSSQAI 464
                ***.**:* .** *:*:***:*  ***     .
```

SeqA	Name	Len(aa)	SeqB	Name	Len(aa)	Score
1	Pfl01_2848	459	2	PA14_18300	464	76
1	Pfl01_2848	459	3	PA3559	464	76
1	Pfl01_2848	459	4	PFL_3050	454	87
1	Pfl01_2848	459	5	PSPPH_2809	464	78
2	PA14_18300	464	3	PA3559	464	99
2	PA14_18300	464	4	PFL_3050	454	77
2	PA14_18300	464	5	PSPPH_2809	464	73
3	PA3559	464	4	PFL_3050	454	77
3	PA3559	464	5	PSPPH_2809	464	73
4	PFL_3050	454	5	PSPPH_2809	464	78

Pfl01_3080 (overlaps *nov8*)

```
Pfl01_3080        MSDILWQPDAKRIARSRMDGFRRFINQRHHLKLDDYPALHQWSIDQREAFWQAIVDFFGI 60
PA14_38690        MPAPLWSPSAERIAASRMEAFRRFVNQRHALGLADYPALHAWSVQRREAFWQAIVDFFEV 60
PA1997            MPAPLWSPSAERIAASRMEAFRRFVNQRHALGLVDYPALHAWSVQRREAFWQAIVDFFEV 60
PFL_3577          MSDLLWQPSAERIGKTRMDAFRRFVNQRHGLNLHDYPALHQWSIDQRESFWQAIVDFFEV 60
PputW619_2720     MNDVLWCPAAAQIEASRMDAFRRWINLRYNLQLNDYAALHGWSVEQRAAFWQSIADYFQV 60
                  *   ** * * :*  :**:.***::* *: * * **.*** **:::* :***:*.*:* :

Pfl01_3080        SFHTQPDAVLRGGLKMPGAEWFPGATLNFAEHLLSRRDDAIAVIAIGENGQRELLTWAEL 120
PA14_38690        RFQQPPRAVLEEGTQMPSARWFPGATLNFAEHLLRRRDDAPALIAVSEDGRREVLSHAEL 120
PA1997            RFQQPPRAVLEEGAQMPSARWFPGATLNFAEHLLRRRDDAPALIAVSEDGRREVLSHAEL 120
PFL_3577          SFHQPAECVLREGPQMPSAQWFPGATLNFAEHLLKRRDDSVAVVAINENGQREQLTHAEL 120
PputW619_2720     LWHTPPSQVLSEGAQMPDARWFAKATLNFAEHLLRRRDDHPAVTAMREDGQRRSLSHAQL 120
                   ::  .  **  * :**.*.**. ********** ****  *: *: *:*:*. *: *:*

Pfl01_3080        AQQVAGFQASLQAAGVLVGDRIAACMPNTWQTLVAMLATTSLGAIWSCSSPDFGTHGVID 180
PA14_38690        ARHVAGLQKRLAALGIGPGDRVAALMPNTWQTVVGMLATASLGATWSSCSPDFGTQGVID 180
PA1997            ARHVAGLQKRLAALGIGPGDRVAALMPNTWQTVVGMLATASLGATWSSCSPDFGTQGVID 180
PFL_3577          AAQVAGLQRSLRAAGVGLGDRVAACMPNTWQTLVGMLASTSLGAVWSSCSPDFGTQGVVD 180
PputW619_2720     AAEVAGLQKAFKNAGIEPGDRIGAVMPNTWETLVAMLACTSLGAVWSTSSPEFGLHGIID 180
                  * .***:*  :   *:  ***:.* *****:*:*.*** :**** ** .**:** :*::*

Pfl01_3080        RFGQIEPKVLITCAGYRYAGKEIDQTVKVNEILEQLPSLQQLIIVPYARPQAHATDYRTP 240
PA14_38690        RFGQIEPRVLIACAGYRYAGKSLDLTAKLNEVLAGLPGLEQLLVVPYDRPQAQPQDYRCQ 240
PA1997            RFGQIEPRVLIACAGYRYAGKSLDLTAKLNEVLAGLPGLEQLLVVPYDRPQAQPQDYRCQ 240
PFL_3577          RFGQIEPKVLITCAGYRYAGKDIDQTAKVNQILERLPSLEQLLIVPYARSQARAEDYRTP 240
PputW619_2720     RFGQIEPKLLIVCAGYQYAGKQIDQVDKINQVCTQLPGLQQLIVVPYTRPHTQADEFSTQ 240
                  *******::**.****:****.:* . *:*::   **.*:**::*** *.:::. ::

Pfl01_3080        ANVTLWDDFYEPGDEPQFVPVPFDHPLYVLYSSGTTGVPKCIVHSTGGVLLQHVKEHGLH 300
PA14_38690        ARVALWDGFYQAEGEPVFTPVPFEQPLYILYSSGTTGVPKCIVHATGGVLLQHLKEHGLH 300
PA1997            AQVALWDGFYQAEGEPVFTPVPFEQPLYILYSSGTTGVPKCIVHATGGVLLQHLKEHGLH 300
PFL_3577          ASVSLWDDFYRPGGEPEFTPVPFAHPLYILYSSGTTGVPKCIVHGTGGVLLQHLKEHGLH 300
PputW619_2720     AKVSLWGDFYQAGGEPTFTPLPFDHPLYILYSSGTTGVPKCIVHSAGGVLLQHLKEHGLH 300
                  * *:**..**.. .** *.*:** :***:***************.:*******:******

Pfl01_3080        VDLGPGDRLFYYTTCGWMMWNWLVSALAVGSAVVLYDGSPFYPDNERLLELLDDEQVSVF 360
PA14_38690        TDLGDGDRLFYYTTCGWMMWNWLASGLAVGASLVLYDGSPFHPGPERLLDLVDAEDIAVF 360
PA1997            TDLGDGDRLFYYTTCGWMMWNWLASGLAVGASLVLYDGSPFHPGPERLLDLIDAEDIAVF 360
PFL_3577          ADLGPGERLFYYTTCGWMMWNWLVSALATGASLVLYDGSPFHPDNEHLMDLIDREAISVF 360
PputW619_2720     NDLKADDVLFYYTTCGWMMWNWLASGLAVGATLVLYDGSPFHPGPERLLDLIDAEGIQAF 360
                   **  .: ***************.*.**.*:::********:*. *:*::*:* * : .*

Pfl01_3080        GTSPKFLATLESSGIKPRESYDLSHLRTLLCTGSALSPQSYDFVYRDFKPDVCLSSMSGG 420
PA14_38690        GASAKYLAALEKAGVRPRHSHRLDSLRTLLSTGSPLAHESFDYVYRELKSDLCLSSISGG 420
PA1997            GASAKYLAALEKAGMRPRHSHRLDSLRTLLSTGSPLAHESFDYVYRELKSDLCLSSISGG 420
PFL_3577          GTSPKYLAALEKAGSRPRQSHRLESLKTLLCTGSPLSPQSYDYVYREIKSELCLASMSGG 420
PputW619_2720     GTSAKYLAALEQEGLEPARSHRLDSLRLILSTGSPLSPHSYDYVYAKIKADLCLASMSGG 420
                  *:*.*:**:**. * .* .*: *. *: :*.***.*: .*:*:** .:*.::**:*:***

Pfl01_3080        TDIVSCFVNGNPMSAVRRGEIMGKSLAMAVEVWNDAGQPVVGEKGELVCTRSFPAVPIGL 480
PA14_38690        TDIVSCFAIGNPVLPVWRGELQCKALGMAVEIWDDDGRRLASGKGELVCTRHFPSIPLGF 480
PA1997            TDIVSCFAIGNPVLPVWRGELQCKALGMAVEIWDDDGRRLASGKGELVCTRHFPSIPLGF 480
PFL_3577          TDIVACFVAGNPIQPVRRGEMQGKGLGMAIEVWNDQGQPVIGEKGELVCTRHFPSIPVGL 480
PputW619_2720     TDIVSCFVLGNPTLPVRRGEIACKGLGMAVQVWNEHGQPVTGEKGELVCTRHFPSMPLGF 480
                  ****:**. ***  .* ***:  *.*.**:::*:: *: : . ******** **::*:*:

Pfl01_3080        WNDPDGEKLRKSYFSQFPGVWAQGDYAEQLPHGGMLIHGRSDAVLNPGGVRIGTAEIYRQ 540
PA14_38690        WNDPDGTRFHDAYFASFPGVWAHGDYAEETAHGGLVIHGRSDAVLNPGGVRIGTAEIYRQ 540
PA1997            WNDPDGTRFHDAYFASFPGVWAHGDYAEETVHGGLVIHGRSDAVLNPGGVRIGTAEIYRQ 540
PFL_3577          WHDPDQSKLKASYFSLFPGVWAQGDYAEQLAEGGWLIHGRSDAVLNPGGVRIGTAEIYRQ 540
PputW619_2720     WNDNDGSRYREAYFSQFPGVWAQGDYAEQLATGGMVIHGRSDAVLNPGGVRIGTAEIYRQ 540
                  *:* *  : : :**: ******:*****:   ** :************************
```

```
Pfl01_3080         VEKVPQVLDSVAIGQQWQDDVRVVLFVHLKDGVTLDDTLQQQIRQVIRANTTPRHVPAKI 600
PA14_38690         VEKVEEVLESIAIGQDWQGDVRVLLFVRLRDGVRLDEPLRERIRQTIRANATPRHVPAKI 600
PA1997             VEKVEEVLESIAIGQDWQGDVRVLLFVRLRDGVRLDEPLRERIRQTIRANATPRHVPAKI 600
PFL_3577           VEKLEQVLESLAIGQSWEQDVRVVLFVRLQDGVELDEALQEHIRQVIRSNTTPRHVPAKI 600
PputW619_2720      VEKVEQVLESVAIGQQWKGDVRVVLFVRLRDGLVLDEPLQQRIRQVIRQYTTPRHVPAVI 600
                   ***: :**:*:****.*: ****:***:*:**: **:.*:::***.**  :******* *

Pfl01_3080         VAVTDIPRTISGKVVELAVRNVVHGEPVKNTDALANPQALEQFRDRPELNS-- 651
PA14_38690         IQVADIPRTLSGKIVELAVRNVIHGRPVKNTDALANPQALELYRDLAELRD-- 651
PA1997             IQVADIPRTLSGKIVELAVRNVIHGRPVKNTDALANPQALELYRDLAELRD-- 651
PFL_3577           LAVTDIPRTISGKIVELAVRNVVHGLPVKNTDALANPEALEQFRDREELRH-- 651
PputW619_2720      LQVSDIPRTISGKLVELAVRNVVHGLPVKNTDALANPEALEFFRDRTELHVQA 653
                   : *:*****:***:********:** ***********:*** :**  **.
```

SeqA	Name	Len(aa)	SeqB	Name	Len(aa)	Score
1	Pfl01_3080	651	2	PA14_38690	651	68
1	Pfl01_3080	651	3	PA1997	651	68
1	Pfl01_3080	651	4	PFL_3577	651	77
1	Pfl01_3080	651	5	PputW619_2720	653	69
2	PA14_38690	651	3	PA1997	651	99
2	PA14_38690	651	4	PFL_3577	651	73
2	PA14_38690	651	5	PputW619_2720	653	70
3	PA1997	651	4	PFL_3577	651	73
3	PA1997	651	5	PputW619_2720	653	70
4	PFL_3577	651	5	PputW619_2720	653	71

Pfl01_3654 (overlaps *nov11*)

```
Pfl01_3654          MSLPSQVRLIEVGPRDGLQNEAQPISVADKVQLVDALSAAGLGYIEVGSFVSPKWVPQMA 60
PA14_38490          MNLPKKVRLVEVGPRDGLQNEKQPIEVADKIRLVDDLSAAGLDYIEVGSFVSPKWVPQMA 60
PA2011              MNLPKKVRLVEVGPRDGLQNEKQPIEVADKIRLVDDLSAAGLDYIEVGSFVSPKWVPQMA 60
PFL_3934            MSLPSHVRLIEVGPRDGLQNEAQPISVEDKVQLVDALAAAGLGYIEVGSFVSPKWVPQMA 60
PputW619_1943       MPLPEKVRLVEVGPRDGLQNEAQPISVADKVRLVDDLTDAGLAYIEVGSFVSPKWVPQMA 60
PSPPH_2629          MSLPQKVNIVEVGPRDGLQNEAQPISIDDKVRLVDDLTAAGLSHIEVGSFVSAKWVPQMA 60
                    * **.:*.::*********** ***.: **::*** *: *** :********.*******

Pfl01_3654          GSAEVFAQIQRKPGVTYGALAPNLRGFEDALAAGVKEVAVFAAASEAFSQRNINCSISES 120
PA14_38490          GSAEVFAGIRQRPGVTYAALAPNLKGFEAALESGVKEVAVFAAASEAFSQRNINCSIKDS 120
PA2011              GSAEVFAGIRQRPGVTYAALAPNLKGFEAALESGVKEVAVFAAASEAFSQRNINCSIKDS 120
PFL_3934            GSAEVFARIQRKPGVVYGALAPNLRGFEDALAAGVKEVAVFAAASQAFSQRNINCSISES 120
PputW619_1943       GSADVFAAIEQRAGVTYAALAPNLRGFEDALAAGVKEVAVFAAASEAFSQRNINCSISES 120
PSPPH_2629          GSAQVFENIQRREGVIYSALAPNLRGFEDALAAGVREVAVFAAATEGFSRRNLNCSISES 120
                    ***:**  *.:: ** *.******:*** ** :**:********::.**:**:****.:*

Pfl01_3654          LARFAPIMESAKQHGVTVRGYVSCVLGCPYEGEVAPEQVAMVARELYAMGCYEVSLGDTI 180
PA14_38490          LERFVPVLEAARQHQVRVRGYISCVLGCPYDGDVDPRQVAWVARELQQMGCYEVSLGDTI 180
PA2011              LERFVPVLEAARQHQVRVRGYISCVLGCPYDGDVDPRQVAWVARELQQMGCYEVSLGDTI 180
PFL_3934            LERFVPIMDAAKQHGISVRGYVSCVLGCPYEGEVAPEQVAMVARELHDMGCYEVSLGDTI 180
PputW619_1943       LNRFEPIMQAARSHGVRVRGYVSCVLGCPYEGRVSAEQVAPVAKALHEMGCYEVSLGDTI 180
PSPPH_2629          LERFAPIMAAARMHGVQVRGYVSCVLGCPYEGSVSPQQVAAVANELYAMGCYEVSLGDTI 180
                    * ** *:: :*: * : ****:********:* * ..*** **. *  ************

Pfl01_3654          GTGTAGATRRLFEVVSKQVPREKLAGHFHDTYGQAMANIYASLLEGIAVFDSSIAGLGGC 240
PA14_38490          GVGTAGATRRLIEAVASEVPRERLAGHFHDTYGQALANIYASLLEGIAVFDSSVAGLGGC 240
PA2011              GVGTAGATRRLIEAVASEVPRERLAGHFHDTYGQALANIYASLLEGIAVFDSSVAGLGGC 240
PFL_3934            GTGTAGATRRMFEVVGAQVPREKLAGHFHDTYGQALANVYASLLEGIAVFDSSIAGLGGC 240
PputW619_1943       GTGTAGDTRRLFEVVSAHVPREQLAGHFHDTYGQALANVYASLLEGISVFDSSVAGLGGC 240
PSPPH_2629          GTGTPGATRALFNAVSAQIPRGKLAGHFHDTYGQALVNIYASLEEGIQVFDSSVAGLGGC 240
                    *.**.* ** :::.*. .:** :************:.*:**** *** *****:******

Pfl01_3654          PYAKGASGNVATEDVVYLLNGLGIETGIDLDALIAAGQQISSVLGRPTGSRVAKARSAQ- 299
PA14_38490          PYAKGATGNVASEDVLYLLNGLEIHTGVDMHALVDAGQRICAVLGKSNGSRAAKALLAKA 300
PA2011              PYAKGATGNVASEDVLYLLNGLEIHTGVDMHALVDAGQRICAVLGKSNGSRAAKALLAKA 300
PFL_3934            PYAKGASGNVATEDVLYLLNGLGIHTGIDMDRLIDAGRQICNVLGRPSGSRVAKARNAS- 299
PputW619_1943       PYAKGATGNIATEDVVYLLQGLGIETGIDLDRLIAAGQRISKVLGRANGSRVARARSAQ- 299
PSPPH_2629          PYAKGASGNVATEDVQYMLQGMGIETGVDLDQVIAAGQRICGVLRRSNGSRVARARLSA- 299
                    ******:**:*:*** *:*:*: *.**:*:. :: **::*. ** :..***.*:*   :
```

SeqA	Name	Len(aa)	SeqB	Name	Len(aa)	Score
1	Pfl01_3654	299	2	PA14_38490	300	77
1	Pfl01_3654	299	3	PA2011	300	77
1	Pfl01_3654	299	4	PFL_3934	299	89
1	Pfl01_3654	299	5	PputW619_1943	299	83
1	Pfl01_3654	299	6	PSPPH_2629	299	78
2	PA14_38490	300	3	PA2011	300	100
2	PA14_38490	300	4	PFL_3934	299	77
2	PA14_38490	300	5	PputW619_1943	299	77
2	PA14_38490	300	6	PSPPH_2629	299	71
3	PA2011	300	4	PFL_3934	299	77
3	PA2011	300	5	PputW619_1943	299	77
3	PA2011	300	6	PSPPH_2629	299	71
4	PFL_3934	299	5	PputW619_1943	299	81
4	PFL_3934	299	6	PSPPH_2629	299	75
5	PputW619_1943	299	6	PSPPH_2629	299	78

Pfl01_3948 (overlaps *nov13*)

```
Pfl01_3948      MIFTPEHEALRRTVRQFVEHEINPHVDEWEKAGRFPIHEIFRKAGDLGLLGISKPEKFGG 60
PA14_26700      MIFTQEHEELRRTVRSFVEREINPNVDQWEKDGRFPIHEIFRKAGELGLLGISKPEKFGG 60
PA2889          MIFTQEHEELRRTVRSFVEREINPNVDQWEKDGRFPIHEIFRKAGELGLLGISKPEKFGG 60
PFL_4197        MIFTQEHEELRRTVRQFVDREINPHVEEWEKAGRFPIHEIFRKAGELGLLGISKPEKFGG 60
Pmen_2692       MIFTQEHEELRRTVRNFVDKEINPHVDAWEKEGRFPIHEIFKKAGELGLLGISKPEKFGG 60
                **** *** ******.**::****:*: *** *********:***:**************

Pfl01_3948      MGLDYSYSIVAAEEFGTIHCGGIPMSIGVQTDMCTPALARFGSDELREEFLRPAITGEQV 120
PA14_26700      MGLDYSYSIVAAEEFGSITCGGIPMAIGVQTDMCTPALARFGSDELREEFLRPAIAGEMV 120
PA2889          MGLDYSYSIVAAEEFGSITCGGIPMAIGVQTDMCTPALARFGSDELREEFLRPAIAGEMV 120
PFL_4197        MGLDYSYSIVAAEEFGTIHCGGIPMSIGVQTDMCTPALARFGSDELRDEFLRPAITGEQV 120
Pmen_2692       MGLDYSYSIVAAEEFGTIHCGGIPMSIGVQTDMCTPALARFGSDELREEFLRPAISGEMV 120
                ****************:* ******:*********************:*******:** *

Pfl01_3948      GCIGVSEVGAGSDVAGLKTTARKDGDDYVINGSKMWITNSPSADFICLLANTSDDKPHIN 180
PA14_26700      GCIGVSEVGAGSDVAGLKTTARKDGDDYVINGSKMWITNSPSADFMCLLANTSDDKPHVN 180
PA2889          GCIGVSEVGAGSDVAGLKTTARKDGDDYVINGSKMWITNSPSADFMCLLANTSDDKPHVN 180
PFL_4197        GCIGVSEVGAGSDVAGLKTHARKDGDDYVINGSKMWITNSPSADFICLLANTSDDKPHVN 180
Pmen_2692       GCIGVSEVGAGSDVAGLKTTARKDGDDYVINGSKMWITNSPSADFICLLANTSDDKPHVN 180
                ******************* **************************:***********:*

Pfl01_3948      KSLIMVPMNTPGISLSSHLDKLGMRSSETAQVFFDNVRVPQRNRIGHEGAGFMMQMLQFQ 240
PA14_26700      KSLIVVPMKTPGITLSPHLDKLGMRSSETAQVFFDNVRVPQRNRIGAEGAGFMMQMLQFQ 240
PA2889          KSLIVVPMKTPGITLSPHLDKLGMRSSETAQVFFDNVRVPQRNRIGAEGAGFMMQMLQFQ 240
PFL_4197        KSLIMVPMNTPGISLSSHLDKLGMRSSETAQVFFDNVRVPQRNRIGHEGAGFMMQMLQFQ 240
Pmen_2692       KSLIMVPMKTPGISVSPHLDKLGMRSSETAQVFFDNVRVPQRYRIGAEGAGFMMQMLQFQ 240
                ****:***:****::*.************************* *** *************

Pfl01_3948      EERLFGAANMIKGLEYCVDSTIEYCKERKTFGNALIDNQVIHFRLAELQTEIECLRALVY 300
PA14_26700      EERLFGAANMIKGLEHCIDVTIEYCKERRTFGQPLIDNQVIHFRMAEMMTEVEALRALVY 300
PA2889          EERLFGAANMIKGLEHCIDVTIEYCKERRTFGQPLIDNQVIHFRMAEMMTEVEALRALVY 300
PFL_4197        EERLFGAANMIKGLEYCVDSTIEYCKERKTFGSPLIDNQVIHFRLAELSTEIECLRALVY 300
Pmen_2692       EERLFGAANMIKGLEHCVDATIDYCKQRHTFGQPLIDNQVIHFRMAELQTEIECLRALVY 300
                ***************:*:* **:***:*:***..**********:**: **:*.******

Pfl01_3948      QATEQYVKGQDVTRLASMAKLKAGRLGREVSDSCLQYWGGMGFMWDNPVARAYRDVRLVS 360
PA14_26700      QATELYVKGRDVTRLASMAKLKAGRLGREVSDACLQYWGGMGFMWDNPVSRAYRDTRLVS 360
PA2889          QATELYVKGRDVTRLASMAKLKAGRLGREVSDACLQYWGGMGFMWDNPVSRAYRDTRLVS 360
PFL_4197        QATEQYVRGQDVTRLASMAKLKAGRLGREVSDSCLQYWGGMGFMWDNPVARAYRDVRLVS 360
Pmen_2692       QATEQYVKGRDVTKLASMAKLKAGRLGREVSDACLQYWGGMGFMWDNPVSRAYRDVRLVS 360
                **** **:*:***:******************:****************:*****.****

Pfl01_3948      IGGGADEIMLGIICKLMGILPGKKK- 385
PA14_26700      IGGGADEIMLGIICKLMGILPGKKKG 386
PA2889          IGGGADEIMLGIICKLMGILPGKKKG 386
PFL_4197        IGGGADEIMLGIICKLMGILPGKKK- 385
Pmen_2692       IGGGADEIMLGIICKLMGILPGKKKG 386
                *************************
```

SeqA	Name	Len(aa)	SeqB	Name	Len(aa)	Score
1	Pfl01_3948	385	2	PA14_26700	386	90
1	Pfl01_3948	385	3	PA2889	386	90
1	Pfl01_3948	385	4	PFL_4197	385	96
1	Pfl01_3948	385	5	Pmen_2692	386	92
2	PA14_26700	386	3	PA2889	386	100
2	PA14_26700	386	4	PFL_4197	385	90
2	PA14_26700	386	5	Pmen_2692	386	92
3	PA2889	386	4	PFL_4197	385	90
3	PA2889	386	5	Pmen_2692	386	92
4	PFL_4197	385	5	Pmen_2692	386	92

Pfl01_4213 (overlaps *nov14*)

```
Pfl01_4213          MRRVVITGLGIVSCLGNDKETVSANLRASRPGIRFNPEYAEMGLRSQVSGSIDLNLEELI 60
PA14_43690          MRRVVITGLGIVSCLGNDKDTVSANLRAGRPGIRFNPSYAEMGLRSHVSGSVDLNLEELI 60
PA1609              MRRVVITGLGIVSCLGNDKDTVSANLRAGRPGIRFNPSYAEMGLRSHVSGSVDLNLEELI 60
PFL_1736            MRRVVITGLGIVSCLGNDKETVSANLRASRPGIRFNPEYAEMGLRSQVSGSIDLNLEELI 60
PputW619_3498       MRRVVITGLGIVSCLGNDKATVTENLRNSRPGIRYNPEYKEQGLRSQVSGSIDLNLEELI 60
PSPPH_1989          MRRVVITGLGIVSCLGNDKETVTANLRANRPGIRFNPEYAEMGLRSQVSGSIDLNLEELI 60
                    ******************* **: *** .*****:**.* * ****:****:********

Pfl01_4213          DRKIYRFVGHAAAYAYLAMKDAIADSGLTEEQVSNPRTGLIAGSGGASTLNQMEALDILR 120
PA14_43690          DRKVFRFMGDAAAYAYLAMEQAIKDSGLTPEQISNPRTGLIAGSGGASTLNQMEAIDTLR 120
PA1609              DRKVFRFMGDAAAYAYLAMEQAIKDSGLTPEQISNPRTGLIAGSGGASTLNQMEAIDTLR 120
PFL_1736            DRKIYRFVGHAAAYAYLAMKDAIADSGLSEEQVSNPRTGLIAGSGGASTLNQMEALDILR 120
PputW619_3498       DRKVYRFVGHAAAYAYLAMQDAIKDAGLTEEQVSNPRTGLVAGSGGASTLNQMEALDTLR 120
PSPPH_1989          DRKIFRFVGHAAAYAYLAMKDAITDSGLTEEQVSNVRTGLIAGSGGASTLNQMEALDTLR 120
                    ***::**:*.*********::** *:**: **:** ****:**************:* **

Pfl01_4213          EKGVKRVGPYRVTRTMSSTVSACLATPFKIKGLNYSIASACATSAHCIGTAMEQIQMGKQ 180
PA14_43690          EKGVKRIGPYRVTRTMGSTVSACLATPFQIKGVNYSISSACATSAHCIGQAMEQIQLGKQ 180
PA1609              EKGVKRIGPYRVTRTMGSTVSACLATPFQIKGVNYSISSACATSAHCIGQAMEQIQLGKQ 180
PFL_1736            EKGVKRVGPYRVTRTMSSTVSACLATPFKIKGLNYSIASACATSAHCIGTAMEQIQMGKQ 180
PputW619_3498       EKGVKRVGPYRVTRTMGSTVSACLATPFKIKGLNYSISSACATSAHCIGTALEQIQWGKQ 180
PSPPH_1989          EKGVKRVGPYRVTRTMGSTVSACLATPFKIKGVNYSISSACATSAHCIGNAVEQIQLGKQ 180
                    ******:*********.***********:***:****:*********** *:**** ***

Pfl01_4213          DIVFAGGGEEEHWSQSFLFDAMGALSSKRNDTPEQASRAYDADRDGFVIAGGGGMVVVEE 240
PA14_43690          DVVFAGGGEEEHWSQSCLFDAMGALSTQYNETPEKASRAYDAKRDGFVIAGGGGMVVVEE 240
PA1609              DVVFAGGGEEEHWSQSCLFDAMGALSTQYNETPEKASRAYDAKRDGFVIAGGGGMVVVEG 240
PFL_1736            DIVFAGGGEEEHWTQSFLFDAMGALSTQYNETPEKASRAYDAKRDGFVIAGGGGMVVVEE 240
PputW619_3498       DIVFAGGGEEEHWSQSFLFDAMGALSTKRNDTPELASRAYDADRDGFVIAGGGGMVVVEE 240
PSPPH_1989          DIVFAGGGEEEHWSQSCLFDAMGALSTQYNETPEKASRAYDAKRDGFVIAGGGGMVVVEE 240
                    *:***********:** *********:: *:*** *******.****************

Pfl01_4213          LEHALARGAKIYAEIVGYGATSDGYDMVAPSGEGAIRCMQQALSTVDTP-IDYLNTHGTS 299
PA14_43690          LEHALKRGAKIYAEIVGYGATSDGYDMVAPSGEGAIRCMQQALATVDAP-IDYLNTHGTS 299
PA1609              LEHALKRGAKIYAEIVGYGATSDGYDMVAPSGEGAIRCMQQALATVDAP-IDYLNTHGTS 299
PFL_1736            LEHALARGAKIYAEIVGYGATSDGYDMVAPSGEGAIRCMEMAMSTVDTP-IDYLNTHGTS 299
PputW619_3498       LEHALARGAKIYAEIVGYGATSDGYDMVAPSGEGAIRCMQQALATVDTP-IDYLNTHGTS 299
PSPPH_1989          LEHALARGAKIYAEIVGYGATSDGYDMVAPSGEGAIRCMQMALGDINSGDIDYINTHGTS 300
                    ***** ********************************: *:. :::  ***:******

Pfl01_4213          TPVGDVAEMKGVREVFGDKAPAISSTKSLSGHSLGAAGVHEAIYCMLMMEGNFIAGSANI 359
PA14_43690          TPVGDVAEIRGVREVFGDKAPAISSTKSLSGHSLGAAGVHEAIYCLLMMEGGFIAGSANI 359
PA1609              TPVGDVAEIRGVREVFGDKAPAISSTKSLSGHSLGAAGVHEAIYCLLMMEGGFIAGSANI 359
PFL_1736            TPVGDVAEMKGVREVFGDKAPAISSTKSLSGHSLGAAGVHEAIYCMLMMEGNFMAGSANI 359
PputW619_3498       TPVGDVAEIKGVRAVFGDKAPKISSTKSLSGHSLGAAGVHEAIYCLLMMENNFIAGSANI 359
PSPPH_1989          TPVGDAKEMEGVREVFGAKAPAISSTKSLSGHSLGAAGVHEAIYCLLMMENNFIAGSANI 360
                    *****. *:.*** *** *** ***********************:****..*:******

Pfl01_4213          DELDPEVADLPVLTKTRENATINTVMSNSFGFGGTNATLVLKRWEGK 406
PA14_43690          DELDPEVADLPILRETRENAKLDTVMSNSFGFGGTNATLVLKRWQG- 405
PA1609              DELDPEVADLPILRETRENAKLDTVMSNSFGFGGTNATLVLKRWQG- 405
PFL_1736            DELDPEVADMPILTKTREDATINTVMSNSFGFGGTNATLVLKRWQGK 406
PputW619_3498       DELDPEVADLPILRKTEENAKIDTVMSNSFGFGGTNATLVLKRWTGK 406
PSPPH_1989          DELDPVVADMPILLKTREDAKLDLVMSNSFGFGGTNATLVLKRWAGK 407
                    ***** ***:*:* :*.*:*.:: ******************** *
```

SeqA	Name	Len(aa)	SeqB	Name	Len(aa)	Score
1	Pfl01_4213	406	2	PA14_43690	405	88
1	Pfl01_4213	406	3	PA1609	405	88
1	Pfl01_4213	406	4	PFL_1736	406	96
1	Pfl01_4213	406	5	PputW619_3498	406	92
1	Pfl01_4213	406	6	PSPPH_1989	407	89
2	PA14_43690	405	3	PA1609	405	99
2	PA14_43690	405	4	PFL_1736	406	88
2	PA14_43690	405	5	PputW619_3498	406	88
2	PA14_43690	405	6	PSPPH_1989	407	88
3	PA1609	405	4	PFL_1736	406	88
3	PA1609	405	5	PputW619_3498	406	88
3	PA1609	405	6	PSPPH_1989	407	88
4	PFL_1736	406	5	PputW619_3498	406	89
4	PFL_1736	406	6	PSPPH_1989	407	90
5	PputW619_3498	406	6	PSPPH_1989	407	88

Pfl01_4382 (overlaps *nov15*)

```
Pfl01_4382      MALNGFGEPGTFLPLSQQLIHSRERPMFDSLSIRLKIVLLSGLCLLGVVALIVGINIYQT 60
PA14_29760      -------------------------MFLRRLSIQWKITLLAGLCLLGVVALLVGLSVYRM 35
PA2654          -------------------------MFLRRLSIQWKITLLAGLCLLGVVALLVGLSVYRM 35
PFL_4630        ---------------MLSIRPFARSPPMFDSLSIRLKIVLLSGLCLLGVVALIVGMNLYQS 46
PFLU4971        -----------------------MSEPMFNTLSIRLKIVLLSGLCLLGVIALVVSINIYNT 38
                                          ::  ***: **.**:*******:**:*.:.:*.

Pfl01_4382      NQNDELVSTSSSKMLTASVQDLLQAKAAEQAVQVQKTFGESLLVITALADQVKDMRVMAA 120
PA14_29760      QHSSVLVKSASTQMLDESARLRLEARGELQALRIQRYFMDAFQYGKGFSRQILFLRDQAQ 95
PA2654          QHSSVLVKSASTQMLDESARLRLEARGELQALRIQRYFMDAFQYGKGFSRQILFLRDQAQ 95
PFL_4630        QQNNQLVNTSSTRMLTAGVEELLQAKAADQAVRVQKTFGESNLVITALADQVRDLRDMAS 106
PFLU4971        TQNDQLVSDSSSRMLTASVEQLLQARAAEQAVQLQKTFGDSLVALTALADQVKDLRNLAA 98
                 :.. **. :*::**  ...  *:*:.  **:::*: * ::    ..:: *:  :*  *

Pfl01_4382      KRSLDAGALREELNQSLKTAFERNSKVLGIWLAFEPNGLDGKDSEFANDAARQSNEAGRF 180
PA14_29760      KRFLDAYDLREDLTRQVRTALAANPEVLGLYVVFEPNALDGKDELFVDQPALGSNDKGRF 155
PA2654          KRFLDAYDLREDLTRQVRTALAANPEVLGLYVVFEPNALDGKDELFVDQPALGSNDKGRF 155
PFL_4630        KRGLEAAALREELNHSLKTAFERNPKVLGIWLAYEPNALDGKDSEFLNDAARASNERGRF 166
PFLU4971        QRGLEPGALREALNQSLKSAFERNNKVLGIWLSFEPNGLDGKDNEFIDDKARVSNEKGRF 158
                :* *:.  *** *.:.:::*:  *  :***::: :***.*****. *  :: *   **: ***

Pfl01_4382      ASYWSRAA-GPSLNTIMVEDDMNKTTLSVSGTPYNSWYTCPRDNKRTCLLDPYADTVGG- 238
PA14_29760      SLYWAQATPGQLESESMVESELADTSSGPSGAAYNAWYTCPKESAQPCVLDPYFDKVGE- 214
PA2654          SLYWAQATPGQLESESMIESELADTSSGPSGAAYNAWYTCPKESGQPCVLDPYFDKVGE- 214
PFL_4630        ATYWSRSG-GQQLNTVMVEDDLTKTTLNLSGTPYNVWYTCPRDSRRTCLLDPYADTIGED 225
PFLU4971        SSYWSRAG-GQGLNTIMVEDDLTKTSLNLSGTPYNIWYTCPRDSRSICLLDPYEDTVAG- 216
                : **:::  *    .   *:*.:: .*: . **:.** *****::.   *:**** *.:.

Pfl01_4382      -KEMLMTTISVPLLVDGKAIGVVGIDIALDALQAAAVNSQRELFNSAGHMLIVSGSGVLG 297
PA14_29760      -RQLLMTSIAFPLELDGKVIGVMGLDINLSNLQALSEQGNRELYDGVGQVGILSPAGLFA 273
PA2654          -RQLLMTSIAFPLELDGKVIGVMGLDINLSNLQALSEQGNRELYDGVGQVGILSPAGLFA 273
PFL_4630        KKQVLMTTISQPLLVDGKVIGVIGVDIALDSLQAAAGESQRFLFNGAGHMMIVAGSGLLA 285
PFLU4971        -KPVLMTTISLPLIVDGKVIGVVGVDIALTALQATTDAAQKNLFNGAAHLEILSSSGLIA 275
                 : :***:*: ** :***.***:*:** *  *** :  .:: *::...:: *:: :*::.

Pfl01_4382      AYSTDATKVGKGITETLGAEGKDILQLLSAGTPKILEQGDLIRAVYPVDPIGNSRAWGVV 357
PA14_29760      GNSRDAGLLGKNLAKADPQHADELLQLLAAGKSQLFNENDDLKVLQPLQPIPGAKPWGVL 333
PA2654          GNSRDAGLLGKNLAKADPQHAGELLQLLAAGKSRLFNENDDLKVLQPLQPIPGAKPWGVL 333
PFL_4630        AVGADASQVGKNINETLGAQGKDILALLAGNQNKVLQQGELIRAVYPFSPIADAKPWGVT 345
PFLU4971        AYSGEPAKVGKNLIDTLGTEGKEIVQLLASDNRMIREQDGTIRAVYPVKPIAEAKSWGVV 335
                . . :.  :**.: .:   .. ::: **:..   : ::.  ::.: *..**  ::.***

Pfl01_4382      IDLPKQVLLADSVKLQAVLDDAQQTGMITALAVAVIAGLIGLLLIWLTASGVTRPINSVA 417
PA14_29760      LEVPKSALLGPALALERQLDDMRREGTWVELGLGLGAAVLGLLVLWLSARGVTRPILGVA 393
PA2654          LEVPKSALLGPALALERQLDDMRREGTWVELGLGLGAAVLGLLVLWLSARGVTRPILGVA 393
PFL_4630        IDLPQQVLLADSVKLQGLLDEAQTSGTVKTVLVAVGAGLLGLLLIWLSASGVTRPINSVA 405
PFLU4971        IKLPKAVMLADNVKLQGVLDEAQAAGTLKALLVAAAAGLLGLLLIWLTATGVTRPINSVA 395
                :.:*: .:*.  : *:  **: :  *    : :. *.::***::**:* ****** .**

Pfl01_4382      EMLKNIASGEGDLTQRLNYTKKDELGELVNWFNRFLDKLQPTIAQIKQSITEARGTADQS 477
PA14_29760      HMLRDIASGEGDLTQRLPHTGRDELGELAGWFNRFLDKLQPIIRDVKVSVRDARSTADQS 453
PA2654          HMLRDIASGEGDLTQRLPHTGRDELGELAGWFNRFLDKLQPIIRDVKVSVRDARSTADQS 453
PFL_4630        QMLKAIASGDGDLTQRLDYSKKDELGELVGWFNRFLDKLQPTIAQIKQSITEARGTADQS 465
PFLU4971        AMLKDIASGEGDLTQRLAYAKKDELGELVNWFNRFLDKLQPTIAQIKQSITEARGTADQS 455
                 **: ****:******* :: :******..*********** * ::* *: :**.*****

Pfl01_4382      SEIARQTSEGMQVQFREIDQVATASNEMSATAHDVANSASNAANAAKGADQSAKDGMSII 537
PA14_29760      AAISSQTSAGMQQQFREIDQVATASHEMTATAQDVARSAAQAADAARGADQATRDGLALI 513
PA2654          AAISSQTSAGMQQQFREIDQVATASHEMTATAQDVARSAAQAADAARGADQATRDGLALI 513
PFL_4630        SEIARQTSEGMQVQFREIDQVATASNEMSATAHDVANSASNAAQAARGADQSAKEGMTII 525
PFLU4971        SAIARQTSEGMQVQFREIDQVATASNEMSATAHDVANSASNAANAARGADQSAREGMQII 515
                : *: *** *** ************:**:***:***.**::**:**:****::::*: :*
```

```
Pfl01_4382      ERSTRDINQLADEVSKAVTEVEELAVNSEQIGSVLEVIRSIAEQTNLLALNAAIEAARAG 597
PA14_29760      DRTTQSIDSLAANLTSAMGQVEQLASSSEEIGSVLEVIRAIAEQTNLLALNAAIEAARAG 573
PA2654          DRTTQSIDSLAANLTSAMGQVEQLASSSEEIGSVLEVIRAIAEQTNLLALNAAIEAARAG 573
PFL_4630        ERSTRDISLLADEVSKAVGEVEALAVSSEQIGSVLEVIRSIAEQTNLLALNAAIEAARAG 585
PFLU4971        EQSTRDITTLAEEVSKAVTEVEALAVNSEQIGSVLEVIRSIAEQTNLLALNAAIEAARAG 575
                :::*:.*  ** :::.*: :** ** .**:*********:********************

Pfl01_4382      ESGRGFAVVADEVRNLAKRTQDSVEEIRVVIERIQTGTRGVVATMHSSQTQAHNNAGQIR 657
PA14_29760      DAGRGFAVVADEVRNLARRTQDSVEQIRGVIEGLQQGTRDVVDAMHGSHRQAQGSVEQVD 633
PA2654          DAGRGFAVVADEVRNLARRTQDSVEQIRGVIEGLQQGTRDVVDAMHGSHRQAQGSVEQVD 633
PFL_4630        ESGRGFAVVADEVRNLAKRTQDSVEEIRVVIERIQSGTRDVVTTMHASQSQAQNNAGQIQ 645
PFLU4971        ESGRGFAVVADEVRNLAKRTQDSVEEIRLVIERIQSGTRGVVATMHSSQSQAQSNAGQIH 635
                ::***************:*******:** *** :* ***.** :**.*: **:... *:

Pfl01_4382      QAVDALGKISDAVTVISDMNLQIASAAEQQSAVAEEVNRNVSAIRTVTETLTEQATESAA 717
PA14_29760      EAVAALQRIGEAVTVINDMNLQIASAAEEQSSVAEEINRNVAAIRDVTESLSSQAEESAQ 693
PA2654          EAVAALQRIGEAVTVINDMNLQIASAAEEQSSVAEEINRNVAAIRDVTESLSSQAEESAQ 693
PFL_4630        QAVQALGKISDAVTVISDMNLQIASAAEQQSAVAEEVNRNVSAIRTVTETLTEQASESAQ 705
PFLU4971        QAVQALGKISDAVTVISDMNLQIASAAEQQSAVAEEVNRNVSAIRTVTETLTGQATESAA 695
                :** ** :*.:*****.***********:**:****:****:*** ***:*: ** ***

Pfl01_4382      ISSQLNALASQQMKLMDQFRV 738
PA14_29760      VSQSLNRLANHQQGLMEQFKA 714
PA2654          VSQSLNRLANHQQGLMEQFKA 714
PFL_4630        VSSQLNALATQQMKLMDQFRV 726
PFLU4971        ISSQLNALASQQMKLMDQFKV 716
                :*..** **.:*  **:**:.
```

SeqA	Name	Len(aa)	SeqB	Name	Len(aa)	Score
1	Pfl01_4382	738	2	PA14_29760	714	52
1	Pfl01_4382	738	3	PA2654	714	52
1	Pfl01_4382	738	4	PFL_4630	726	80
1	Pfl01_4382	738	5	PFLU4971	716	80
2	PA14_29760	714	3	PA2654	714	99
2	PA14_29760	714	4	PFL_4630	726	54
2	PA14_29760	714	5	PFLU4971	716	53
3	PA2654	714	4	PFL_4630	726	54
3	PA2654	714	5	PFLU4971	716	53
4	PFL_4630	726	5	PFLU4971	716	78
