# Supplementary material for: Proteomic Detection of Non-Annotated Protein-Coding Genes in Pseudomonas fluorescens Pf0-1
Source: PLoS One. 2009 Dec 24;4(12):e8455. doi: 10.1371/journal.pone.0008455 (PMC2794547; doi:10.1371/journal.pone.0008455)
Supplement: Figure S2 — Alignment of Nov proteins with putative proteins identified using TBlastN. Alignments were generated with ClustalW2 (http://www.ebi.ac.uk/Tools/clustalw2/index.html ). Full start codon to stop codon sequences in each genome were determined by examining the sequence in the Artemis genome browser (http://www.sanger.ac.uk/Software/Artemis/). (0.13 MB PDF) [file pone.0008455.s002.pdf]

Figure S2. Alignment of Nov proteins with putative proteins identified using TBlastN. Alignments were generated with ClustalW2 (http://www.ebi.ac.uk/Tools/clustalw2/index.html). Full start codon to stop codon sequences in each genome were determined by examining the sequence in the Artemis genome browser (http://www.sanger.ac.uk/Software/Artemis/).

Colors in alignments:
RED = AVFPMILW (small+ hydrophobic (incl.aromatic -Y))
BLUE = DE (Acidic)
MAGENTA = RK (Basic)
GREEN = STYHCNGQ (Hydroxyl + Amine + Basic - Q)

Consensus symbols in the alignments:
* = residues or nucleotides in that column are identical in all sequences in the alignment.
: = conserved substitutions.
. = semi-conserved substitutions.

Nov1
P. fluorescens Pf-5 ORF is the complement of Bases 798825..799727

```
nov1            VGRGQRRRHRSAFAGADFRAVLHHQTGGRRHWPGLVGELRHRREHGRTPERAQRRRRRPV 60
Pf-5            VGRGQWWRHRPAPAGADLRAVLHHQAGGHRHRARAVGELWHRAEHGGPPERQQQPGRRAL 60
                *****  ***.* ****:*******:**:** .  **** ** *** .*** *:  **.:

nov1            LHRIADRPRRSDHQRRPPRLTT-EIGADIDLIEVDPQGHQQRVDEGVEQRTEHRIDGGDQ 119
Pf-5            LHRTADRRGGSDHQVGLAGLPAGEVGPHFHVHQVDPQPRQQVIQQRVQQRAQEPGDQRRE 120
                *** ***   ****   . *.: *:*..:.: :**** :** ::: *:**::.  *   :

nov1            QGLHGAQNRPQSTDQTAR-----RAMHVEVEVVQGVAQAADHVAG--------------- 159
Pf-5            QVADPADGRSQPRDNGPQRVVVDRLVDDQVDAGQGARQAVDDVRGRGRRLVSRGLVDVGR 180
                *  . *:.*.*. *: .:     * :. :*:. **. **.*.* *

nov1            ----------------------------AVAVIERRLVDLRQT----------------- 174
Pf-5            YRKRRRRAGEHQVLSILRHAGVDHHRDAAARVVHHRVAAPRQDQELVAGAYAQVHQRQRL 240
                                            *. *:.:*:.   **

nov1            ----GSRRCGVDMGDVGVLADG----GIG------------------------------- 195
Pf-5            HHGLGGRRKGIGRVDLADLQVGDGTDGMGGQALGPAVVHRAAVVGDIGNRAPRVQAQVDL 300
                    *.** *:.  *:. *  *    *:*

nov1            -
Pf-5            R 301

SeqA Name    Len(aa)   SeqB Name    Len(aa)   Score
===============================================
1    nov1    195       2    Pf-5    301       43
===============================================
```

Nov2

Photorhabdus asymbiotica ATCC43949 bases 1393742 to 1393996
Yersinia pestis D182038 bases 4332492 to 4332238
Escherichia coli strain J plasmid ColE9-J bases 3807 to 4067; gene is ceiI.

```
nov2              MKFKNKFEEYTEAEYVALINRLVEGDYSSEKEHDAIVDNIVKTSEHPNGTDVLYYPEEGT 60
Photorhabdus      MKLKNKLEEYTENEFIDLISRIIS-DEGTEKEQDTLLENFIKITEHPSGTDLIYYPENGE 59
Yersinia          MELKEKYEDYTEHEFLEFIRNICEVNTDSQSLHSSWVRHFTKITEHPSGSDLIYYPEDGA 60
Escherichia       MELKHSISDYTEAEFLQLVTTICNADTSSEEELVKLVTHFEEMTEHPSGSDLIYYPKEGD 60
                  *::*.. .:*** *:: ::  : . : .::.     : :: : :***.*:*::***::*

nov2              DDSPAGVLETIKAWRAANGKPGFKNEE 87
Photorhabdus      NDSPEGILKTIKEWRAKNGKPGFKE-- 84
Yersinia          DDSPEGILELVKKWRAENGKPGFKK-- 85
Escherichia       DDSPSGIVNTVKQWRAANGKSGFKQG- 86
                  :*** *::: :* *** ***.***:
```

SeqA	Name	Len(aa)	SeqB	Name	Len(aa)	Score
1	nov2	87	2	Photorhabdus	84	58
1	nov2	87	3	Yersinia	85	49
1	nov2	87	4	Escherichia	86	47
2	Photorhabdus	84	3	Yersinia	85	55
2	Photorhabdus	84	4	Escherichia	86	52
3	Yersinia	85	4	Escherichia	86	57

Nov3 (note that full sequence from P. putida is not shown)

P. fluorescens SBW25 bases 5525882 to 5526268
P. putida KT2440 (P. p KT2440) complement of bases 1184848 to 1188960

```
nov3            MAQLKSRHYRANQLIANDKLDLPLLDPLNLPSLTNERLTPVQRPKLTRAKHLPSTVVIVT 60
SBW25           MAKLECSHDSHNQLIRHHQLNLPFLNHFNLAHLPNKRLAAVQRAELALAQHLPGAAVVVA 60
P. p KT2440     VALLERSDDLHNQLVSHHQLHLPLFHRLDLARLPEERLAAVQRAELALAKGLPGAVVIVT 60
                :* *:  .   ***: :.:*.**::. ::*. *.::**:.***.:*: *: **.:.*:*:

nov3            DPPTTGSEYSLLDHLDMTVGNKKRELVHSA------------------------------ 90
SBW25           DAPAASCEDGGFHRLHVAVGHEEAELVHGLSSP--------------------------- 93
P. p KT2440     DAPAARGEHSGLDHFDMAGGDEEGELVHALVHPHPRVAEHTHQRSIFLPLVRAPGVLHSA 120
                *.*::  * . :.::.:: *.:: ****.

nov3            ------------------------------------------------------------
SBW25           -----------------------TR----------------------------------- 95
P. p KT2440     EHTLRVRHHDGHAAVAAGQAGDTTRRAVRVGRVGFGDLAMVVDVAQGHATGHIGFQQCFF 180


nov3            ------------------------------------------------------------
SBW25           ------------------------------------------------------------
P. p KT2440     AGELGMAFAVGDGDRHARTGHALQEDRVGLLHFDHGHAGLELLRAVAHEVRPVLGARDQL 240


nov3            ------------------------------------------------------------
SBW25           --------------------------GRCGTCASRRRPRPTLPGATS------------- 116
P. p KT2440     VQVGHHLATVAHAQGEAVGTLEEGLEGVTGTGVEQRRLGPAFASAQHVTVREAATGDQAL 300


nov3            ------------------------------------------------------------
SBW25           ---------------------------------------------------SARRG---- 121
P. p KT2440     EGFEVDTASENIAHVHVDGGEAGAVEGSSHLDLAVDALLAQDGHLRAHALLDVRRGDVVV 360


nov3            ------------------------------------------------------------
SBW25           ----------------------IHAPGAAS------------------------------ 129
P. p KT2440     DVEAQLHRQARVVFLEQQVELLISAVGVVAQALDLVAGFSPLALQHATVIVEQHFAVQAD 420
```

SeqA	Name	Len(aa)	SeqB	Name	Len(aa)	Score
1	nov3	90	2	SBW25	129	42
1	nov3	90	3	P. p	1371	46
2	SBW25	129	3	P. p	1371	48

Nov 4 – No significant hits

Nov 5 – No significant hits

Nov 6 – No significant hits

Nov 7 (note the stop codon in SBW25)

P. fluorescens SBW25 bases 3312166 to 3312351
P. fluorescens Pf-5 complement of bases 3558023 to 3558211

```
nov7            -------------------------------LTKALSRCARKVSPVPTGTVDFSTRISGF 29
SBW25           MRSGRRQSATAEPSLRNSGLETISKSKCRPARCKAFSICARRVSPVPTGTVDFSTRITGS 60
Pf-5            --------VTAEPSLRNSGLETISNCSARP--SRTFSMWAFKVSPVPTGTVDFSTRITGC 50
                                                 :::*  * :***************:*

nov7            SRCRATASPTDST 42
SBW25           LR*RATASPTDST 73
Pf-5            SRWRATASPTDST 63
                 * **********
```

SeqA	Name	Len(aa)	SeqB	Name	Len(aa)	Score
1	nov7	42	2	SBW25	73	78
1	nov7	42	3	Pf-5	63	73
2	SBW25	73	3	Pf-5	63	77

Nov8

P. fluorescens SBW25 bases 2894607 to 2895233

```
nov8            VHHIAHGQLHHFAADGARNVGDRDDLRRHMARCGVGTDHLADLLLQRVIQRYAVLEVHEQ 60
SBW25           VHHIAHRQFNDLAADGAGDIGDSEDLRRYVAGCGVGADHLADLLLKGFIQLDTVIEAHEQ 60
                ****** *::.:***** ::** :****::* ****:********: .**  :*:*.***

nov8            HHAHIVLPLLADGDAVQHLRDFLHLTVDFRRADAHAAGVEHGVGAAVNQHPAVGQLFGVV 120
SBW25           HHPYVILPALANGQALLHLRDFFHLAINLCRADAHATGVEHRVGTPVNQQAAVGALLGIV 120
                **.:::** **:*:*: *****:**:::: ******:**** **:.***:.*** *:*:*

nov8            TLGPDAGELAEIGFAQFLAIGIVPESDRHRRK---------------------------- 152
SBW25           ALGPDTGELAEIRGAQLLAIRVVPQADGHGRKVPGAHQFAFFADQRLALVVPHFNRHCQA 180
                :****:******  **:*** :**::* * **

nov8            -----------------------------
SBW25           LALHFAAPHRQHWVADHKAGNDIGATGHG 209
```

SeqA	Name	Len(aa)	SeqB	Name	Len(aa)	Score
1	nov8	152	2	SBW25	209	62

Nov9 - No significant hits

Nov10

P. fluorescens Pf-5 bases 2411768 to 2411950

```
nov10           MSMAMLNKMHMNGYDVLSVNNGPWRVCTQGDRLASFASREEALAYAAALPGYKSRARGRK 60
Pf-5            MSTPLLNKLQINGYQVLSVNHGPWKVCTPGDRLATFGTREEAMAFAAALPARRQRPAATG 60
                ** .:***:::***:*****:***:*** *****:*.:****:*:*****. :.*. .

nov10           AD 62
Pf-5            G- 61
```

SeqA	Name	Len(aa)	SeqB	Name	Len(aa)	Score
1	nov10	62	2	Pf-5	61	59

Nov11

P. fluorescens SBW25 complement of bases 4286966 to 4287352

```
nov11           VLFRRFHDRREPRQAFADRAIDVALRKRFGRSGKHRDFLHSRRKCIFKTAQVRRQCTIGY 60
SBW25           VLPGRRHDRRKAFQAFADGTVDIALGKRLGRRRKHRHFLHAGCQCVLETAQVRRQGTVGH 60
                **  * ****:. ***** ::*:** **:**  ***.***:  :*:::******* *:*:

nov11           AGLALDLREHLGGTGHLRHPLGRDETADFYIAETGGAQRVDQLHLVGDADGLGFVLQAVT 120
SBW25           AGPALDLREYLGRAGHLRHPLGRDETADFDIAQAGSAECVHQPDFVRHADRLGFVLQAVA 120
                ** ******:** :*************** **::*.*: *.* .:* .** ********:

nov11           RADFDQAYLGGEGHGVDLLFEFLRNPVWDLFRLLN 155
SBW25           RADFHQAYV-------------------------- 129
                ****.***:
```

SeqA	Name	Len(aa)	SeqB	Name	Len(aa)	Score
1	nov11	155	2	SBW25	129	65

Nov12 - No significant hits

Nov13

P. fluorescens Pf-5 Bases 4883762 to 4885246
P. fluorescens SBW25 Bases 4862082..4863530

```
nov13           VDHQLAERRLHLPAGQHLGRQAAHQQIADHGADEHAGYQPQFAPGQARHAQLGNRPGVFR 60
Pf-5            VDHQLPQRRLHLPAGQHFGRQAPRQQVADHGADEHPGHQPQLAPGQARHAQLGNRPGVLR 60
SWB25           VDHQLTQRRLHLPAGQHLRRQAAHQQIADHGADEHPRHQRQPAPGKTRHAQLRDRPGVLR 60
                *****.:**********: ***.:**:********. :* * ***::***** :****:*

nov13           QRARAPAQSHRPRRRRVHDADAAVPGGTPVRCGQHDQGPGILRRQHHRVLQGAQDLRQRA 120
Pf-5            QCAGASTQPHRPRRRRFHDADAAVPGGTAVRRGEHDQGPGVLRGQHHRVLQGAQDLRQPA 120
SWB25           RRARAAAQPHRPRRRGVHDADAAVPGRTPVRRGQHDQGPGALHRHHHRILQGAPDLRQGA 120
                : * *.:*.****** .********* *.** *:****** *: :***:**** **** *

nov13           DRQSGDSLPPRRVADRNRMPAGAGLSGHRAIRQRPGRHPTGVDGQAQGRTSRSGSQRQLP 180
Pf-5            DRQPGDPLPPGRAVHRDRVPAGPGLPGHRAVCTRPGRDPAGFHGQAQGRAPGPRGQRQLP 180
SWB25           DRQPGHPLSPGRTLHRNRMPARAGLPGHRAIHQRPGRDSPGLHGQAQGRAPGPRSQRQLP 180
                ***.*..*.* *. .*:*:** .**.****:  ****...*..******:. . .*****

nov13           AILGRHGLHVGQPGGPRLPRRAAGIDWRRRR----------------------------- 211
Pf-5            AILGRHGLHVGQPGGPGLSRCAPGVHRRRCRRDHAGDHLQAHGHPAGEKEMNPLPACQTL 240
SWB25           AILGRHGLHVGQPRRPRLSRCAPGLDRRRRRRNHAGHPLQTDGHLAWEKAMN------TL 234
                *************  * *.* *.*:. ** *

nov13           ------------------------------------------------------------
Pf-5            LLEPRNGVLFISLNRPQNRNAMSLQMVAELRTVMAAVRDRRSIRALVLRGTGEHFCAGGD 300
SWB25           LLEPHNGVLHITLNRPENRNAMSLEMVNELRTVLAQLDS--QVRALVISGAGGHFCAGAD 292


nov13           ------------------------------------------------------------
Pf-5            LKDMAGARAHGSAAYRELNRAFGALLEEAQHLPQVLVCVLQGAVLGGGFGLACVSDIAIA 360
SWB25           VKDLVTAGNQ----LQALNRAFGTLLQEVEAVPQVVIVVLQGAVLGGGFGLACVSDIAIA 348


nov13           ------------------------------------------------------------
Pf-5            EHSAQFGMPETSLGLLPAQIAPFVVQRIGLAQARRLALTAARFDGAQAQRLGLVHFVEND 420
SWB25           DHQAQFGLPETSLGLLPAQIAPFVVKRIGLTQARRLALTAARFDGLEAERLGVVHFTERD 408


nov13           ------------------------------------------------------------
Pf-5            AKALHERLEQQLSLVLRCAPGANAATKALLLASVDQPQGPLLDQAANDFANAVLGEEGVE 480
SWB25           PQALAERLDDVLGQVLRCAPGANGRTKSLLLASVDEPLEAVLDRGAQWFAEAVRSEEGIE 468


nov13           ---------------
Pf-5            GTLAFVQKRKPAWAN 495
SWB25           GTQAFVQKRKPRWCK 483
```

SeqA	Name	Len(aa)	SeqB	Name	Len(aa)	Score
1	nov13	211	2	Pf-5	495	74
1	nov13	211	3	SWB25	483	74
2	Pf-5	495	3	SWB25	483	72

Nov14

P. fluorescens Pf-5 bases 1942080..1942268

```
nov14           LHDKRFILAPLKMPFSKRKKTPRTGRGVFMCGSAGGNYLPSQRFSTRVALVPPKPKLLLI 60
Pf-5            --------------------------------------LPCQRFRTRVALVPPKPKLLLI 22
                                                      **.*** ***************

nov14           TVLMVAFSRVLVSTGRSATSGSSSSMLAEPAMKLPSIISMQ 101
Pf-5            TVLMVASSRVLVRIGISATSGSSSSMLAEPAMKLPSIISMQ 63
                ****** *****  * *************************
```

```
SeqA Name     Len(aa)   SeqB Name     Len(aa)   Score
==================================================
1    nov14    101       2    Pf-5     63        90
==================================================
```

Nov15

P. fluorescens Pf-5 complement of bases 5341606..5343108
P. aeruginosa PA14 Bases 2576991..2578589

```
nov15           VGGGAHLVGGGCHLIDLAELHLHAFAGLAGDFRRLIGGATGLGDALFDLGDGRLQLVEEA 60
Pf-5            VGSGAHFVGGGGHLIDFAELHLHAFTGLAGDFRRLVGGAPGFGDALFDLGDGRLQLVEEA 60
PA14            LGSGGHLVGCGGDLIDLAELLLHAGAGLAGNRRRLVGGTARIAHRDLHVADDRLQLVEET 60
                :*.*.*:** * .***:*** *** :****: ***:**:. :..  :.:.*.*******:

nov15           VEPVHQFAQFVFLGVVQALGQVAFAAGDVLQHFGDAVDRAGHAGGGEPDQQQTDQAGDHG 120
Pf-5            VEPAHQFAEFVFLAVVQALGQVAVAAGDGLEHLRHAVDRAGHARGTEPDQQQAQQAGADG 120
PA14            VEPTGQFAQLVAPGVRQTLGQVAFAAGDVAQHVGDAEDRPGHAAGAQPQHQQAEHRGAEA 120
                ***. ***::*  .* *:*****.****  :*. .* **.*** * :*::**::: * ..

nov15           HRQRGNHAGLLGIVEHCLELDRVGQQHLFRQVDDHAPGPGVANRIDRVHGADQVALFENL 180
Pf-5            HQYGFHRAAGLGLIQQALQFYRVCQQHLLRQVDGDAPGLGIGNRAEGVDRADQFALLQDL 180
PA14            EAQFDPGAFAAHVVQLPLQRQGRAEQGALRHFEEHAPGLGAGNRLQRLQHLEVVVLVEQL 180
                .      *    :::  *:     :*  :*:.: .*** * .** : :.  : ..*.::*

nov15           RRAGAQQLQDVLAFGTEGFGDAFADFGCVGAVGAQHAAAGDDQHVSGTVEQLTLGIDGRR 240
Pf-5            VLVAGEQREDILALGAEGFVDVLADLAGIGAHGRQQAAAGDDHHVPGTVEQKTLGFAGRG 240
PA14            ALAGGQQLQEFVGVLRIRLGQVLAEQASVAAVAGEQPGRAEDADLANAVVEFAVALFAER 240
                  ...:* ::.:..    : :.:*: . :.* . ::.. .:* .:..:* : ::.: ..

nov15           LQRIQGNVDADHADGLAVHQQRYGNGGHEH--FLAAHGIGVRIKQAGALVVARAGVPAVV 298
Pf-5            LQGIQGDVDANHPDDLAVHQQGLGDGGHQHLFLVLADGVGVGVQQAGTAAVAGAGVPDVV 300
PA14            LQIAQVDVQAHHADHLAIQFEGEGDAGHQQ--LALADLVEVGIEHAGLSAFLRAGVPGVV 298
                **  * :*:*.*.* **:: :  *:.**::  :  *. : * :::**  ..  **** **

nov15           RRAADAEGGLVHVVFDHDR-VQRRTRSTAPVTGETPRLVGLTRGIVGEFAVLAIQSVRFE 357
Pf-5            GGAAEVEGSLGQVVFHHHG-VQLLAPGSAPVGGEAPAFVGSTGCVIEKFAVLAIQGVGFV 359
PA14            GRAAGPAAGVGEFGLDHRLGLQLSRRGLRPVQGEASLVVAAQGGLVDEQFVLAVQCVGFE 358
                  **   ..: .. :.*   :*    .  **  **:. .*.    :: :  ***:* * *

nov15           RQPDAEHLAVAFEGGFQALVQLFTQGAGVQRTLGSHDAHVLDLVGQGGDHQQALAEGFLH 417
Pf-5            GQPDAEHFGIALEGGLQAVVELFAQCRSFQAALARHVTQVAYLVRQGGDHQVAFTEGLLH 419
PA14            DHVQAEHLGIGGERGTHLAGQVFAQVVGVEEALLRLVAEEQDLPGETLAVLEGIHEIALD 418
                 : :***:.:. * * :   ::*:*  ..: :*    :.   *  :      .: *  *.

nov15           LHRLFGSLGLQQVLHTGGEHLAAGSADQFVVLVGLVNIDANDQRDHTEQAQAGQQDDFQA 477
Pf-5            PYCLVGGLGLQQLFDPGGEHPRAGGVDQLVVLLALVQVHADDQRHYAQQAQSGQQHYFQA 479
PA14            AQRLQFPAGFQAQARRFVEHLRTGAFHQYAAVLHAVDRKADQQGDHAEQAEACQQGDLPL 478
                   *    *:*       **  :*. .* ..::  *: .*::* .:::**:: **  :

nov15           NGQR-VEHGTFSRMDELLAKRQEGARFAKSIQRHSMLIGAGRTGMNHKPTSGRG--- 530
Pf-5            NGER-VEHG------------------GRSRERT-----DGKHGIG----------- 501
PA14            DGQAAKEHGRGPCTKKLRGPK--AYRQGSPILDDGAFAGAGLPSCGESHARTVHNGL 533
                :*:   ***                  . .          *  . .
```

SeqA	Name	Len(aa)	SeqB	Name	Len(aa)	Score
1	nov15	530	2	Pf-5	501	59
1	nov15	530	3	PA14	533	36
2	Pf-5	501	3	PA14	533	37

Nov16

P. fluorescens Pf-5 PFL_5052; Bases 5812292..5812516
P. putida KT2440 complement of Bases 919306..919527

```
Nov16           MSNSMGIASAFVLSSLILSPLAMAEESQTFVAQNAARAHAFEQHQAEVMAKAQDATQAPQ 60
Pf-5            MSNSMGIASAFVLSSLFVAPMAMAEESQGFVARNAARAAAFEQAQIAQAAQQNTQQAQK 60
KT2440          MSNSMGIASVFVLSSLLLSPLAMAEESPAFVARNAEIAAVHQEARDAVAANKADTVKDAK 60
                *********.******:::*:******  ***:**  * ..:: :    *:  :: :  :

Nov16           AATSQAQA-SEKDS 73
Pf-5            ITAEQSKSDTQKDS 74
KT2440          QTASKVSVDTSADS 74
                 ::.: .  :. **
```

SeqA	Name	Len(aa)	SeqB	Name	Len(aa)	Score
1	Nov16	73	2	Pf-5	74	57
1	Nov16	73	3	KT2440	74	47
2	Pf-5	74	3	KT2440	74	56
