# Supplementary material for: Proteomic Detection of Non-Annotated Protein-Coding Genes in Pseudomonas fluorescens Pf0-1
Source: PLoS One. 2009 Dec 24;4(12):e8455. doi: 10.1371/journal.pone.0008455 (PMC2794547; doi:10.1371/journal.pone.0008455)
Supplement: Table S1 — Primers used in RT-PCR (0.06 MB PDF) [file pone.0008455.s003.pdf]

Table S1. Primers used in RT-PCR

Locus	RT/reverse primer (5'-3')	Forward Primer (5'-3')
nov1	TCGGCCAGAACGCCAACATC	GAGCACCGTATTGATGGTGG
nov2	AAACTCCTGCCGGACTGTCC	TGAGGAATATACGGAAGCCG
nov3	AAGGAGGCTGTATTCGCTGC	CCACTACCGAGCGAATCAAC
nov4	TCTACCATTTTGCTGAGGGC	TACAGGGCACTGGATTGACG
nov5	GACCTAACAGTCCCATCCCC	GTGAAAGTCCCAGGCCCTC
nov6	GTTCAGTCCGATGCCTTGCG	TGGTGATATCGCCGTATTGC
nov7	TGTGCTGTCGGTGGGCGATG	ATCCAGATGCGCACGCAAAG
nov8	TGACTACGCCGAACAACTGC	GTGGTGTTGGCACGGATCAC
nov9	ACTTGTCCGGATCCTCGTCC	ATTCCCCAGTGAAGAGCAGG
nov10	GGCTTTTGTACCCCGGCAGC	GCACATGAATGGCTACGACG
nov11	CAAGTGGGTGCCGCAGATGG	TTCCATGATCGGCGCGAACC
nov12	GCGTGCTGAGGAAAGGGGAG	GAGGAAAACAGCTTCGTCGG
nov14	GCATGCTGATGATGGAAGG	ATTACTTGCCTTCCCAGCG
nov15	ACCTGCCGAAACAAGTGCT	CTCGGTGATGCTCTGTTTGA
nov16	TCTCAGAAGCTTGAGCCTGC	AATCCCAGACGTTTGTAGCG
